# microbetag: simplifying microbial network interpretation through annotation, enrichment tests, and metabolic complementarity analysis

**DOI:** 10.1186/s13059-025-03769-2

**Published:** 2025-09-22

**Authors:** Haris Zafeiropoulos, Ermis Ioannis Michail Delopoulos, Andi Erega, Aline Schneider, Annelies Geirnaert, John Morris, Karoline Faust

**Affiliations:** 1 Department of Microbiology, Immunology and Transplantation, Rega Institute for Medical Research, Laboratory of Molecular Bacteriology, KU Leuven, Herestraat 49, Leuven, 3000 Belgium; 2 Institute of Food, Nutrition and Health, Laboratory of Food Biotechnology, ETH Zurich, Schmelzbergstrasse 7, Zurich, 8092 Switzerland; 3 Resource for Biocomputing, Visualization, and Informatics, University of California San Francisco, 16th Street, San Francisco, CA 94143 USA

**Keywords:** Microbial associations, Enrichment analysis, Data integration, Pathway complementarity, Seed set, Phenotypic traits

## Abstract

Microbial co-occurrence network inference is often hindered by low accuracy and tool dependency. We introduce *microbetag*, a comprehensive software ecosystem designed to annotate microbial networks. Nodes, representing taxa, are enriched with phenotypic traits, while edges are enhanced with metabolic complementarities, highlighting potential cross-feeding relationships. *microbetag*’s online version relies on *microbetagDB*, a database of 34,608 annotated representative genomes. *microbetag* can be applied to custom (metagenome-assembled) genomes via its stand-alone version. *MGG*, a Cytoscape app designed to support *microbetag*, offers a streamlined, user-friendly interface for network retrieval and visualization. *microbetag* effectively identified known metabolic interactions and serves as a robust hypothesis-generating tool.

**Supplementary Information:**

The online version contains supplementary material available at 10.1186/s13059-025-03769-2.

## Background

Most microbial species live in communities and most natural microbial communities consist of hundreds or even thousands of species. Each species exhibits a unique repertoire of biochemical reactions and adapts to various niches, each with specific nutrient and environmental requirements. Depending on the net fitness effects that result for the taxa involved, interactions range from cooperation, competition, parasitism, commensalism, and amensalism [1]. In addition to other mechanisms, microorganisms can interact by competing for or exchanging metabolites. The latter interaction mechanism can involve either one-way (unidirectional) or two-way (bidirectional) exchanges of metabolites.

High-throughput sequencing has provided insight into the diversity and composition of microbial communities. Uncultivated species can now be detected, and their traits can be predicted based on their genomic information [2]. Moreover, the composition of thousands of microbiome samples is now accessible, allowing for the inference of associations across large sets of samples. A widely used approach to extract such patterns is the creation of microbial co-occurrence (i.e., association) networks based on microbial sequencing data (amplicon and/or shotgun) [3, 4]. Several approaches are available for co-occurrence network inference based on the assessment of similarity, dissimilarity, and/or correlation (e.g., CoNet [5], SparCC [6]) and conditional dependency identification, which allows reducing the number of indirect edges (e.g., SpiecEasi [7], FlashWeave [8]). Nevertheless, microbial network inference encounters various challenges [9, 10]. Each inference approach comes with its own assumptions and parameter settings, leading to variations in network structure. The choice of the association measure and data preprocessing techniques as well as the handling of sparsity and zero inflation all influence the resulting network. As a consequence, the result of network construction is tool-dependent [11, 12]. Moreover, microbial network inference inherits the challenges of sequencing data and analysis (e.g., sampling scale, compositionality, tuning of parameters linked to sequencing data processing) and the returned network is often a “hairball” of densely interconnected taxa. Thus, additional analysis is necessary to generate testable hypotheses [10].

The comparison of interactions predicted by microbial networks with a collection of known interactions has underscored their low accuracy for this task [11, 13]. Data integration has been suggested to help interpret edges in microbial networks [10]. In addition, clusters in microbial networks have been demonstrated to detect key drivers of community composition [14] and several algorithms and implementations are available to identify them (e.g. [15]). However, data integration approaches available for microbial networks are so far limited.

Metabolic networks are comprehensive representations, in a mathematical form, of biochemical reactions occurring within an organism [16–18]. A metabolic network can be considered as a knowledge-base of its corresponding strain, capable of integrating genomic, regulatory, and phenotypic information. Over the last decade, (semi-) automated approaches support the fast generation of such reconstructions (e.g., [19, 20]). A key concept for the topological analysis of such metabolic networks is the *seed set*. Based on the original definition, a seed set is “the minimal subset of the occurring compounds that cannot be synthesized from other compounds in the network (and hence are exogenously acquired) and whose existence permits the production of all other compounds in the network” [21]. Seeds are a useful proxy for the essential nutrients of an organism [21, 22], and based on the seed concept, several graph theory-based metrics (indices) have been described to predict species interactions directly from their metabolic networks’ topologies [23–26]. As Lam et al. [27] highlight in their study, seed sets “represent a baseline of metabolites that in theory enable a given bacterium to produce any metabolite in their predicted metabolic network.”

Seed and non-seed compound sets, as defined above, can be used to compute complementarity and overlap indices. Metabolic *complementarity* between two species reflects their potential for cooperation through cross-feeding. In contrast, metabolic *competition* refers to the metabolic overlap between two species leading to exploitative competition. The examination of such indices can indicate metabolic interactions that may drive the patterns observed in co-occurrence networks.

To explore whether a species may benefit from a partner, it is helpful to move from the network to the pathway level and check whether their pathways complement each other. For this, we rely on a naive approach that enumerates all possible complements between a pair of species based on their KEGG ORTHOLOGY (KOs) annotations and the KEGG MODULES database [28].

Here, we present *microbetag*, a software ecosystem for microbial network annotation that exploits several sources of information to enhance the confidence in the associations suggested by the network, thereby generating hypotheses for further investigation both at the taxon pair and the community level. *microbetag* serves as a comprehensive platform that provides information about taxa along with their potential metabolic interactions from multiple channels (see “Methods”). The key concept here is the reverse ecology approach [29]. Reverse ecology leverages genomics to explore community ecology with no a priori assumptions about the taxa involved. The reverse ecology framework enables the prediction of ecological traits for less-understood microorganisms and their interactions with others [30]. *microbetag* annotates a user’s co-occurrence network by integrating phenotypic traits of the taxa present in the network (nodes) and by mapping potential metabolic interactions onto their associations (edges). *microbetag* is accompanied by a graphical user interface (GUI) implemented as a Cytoscape [31] app [32] providing a user-friendly environment to investigate annotations in a straightforward way. Its online version depends on precalculated annotations for ~ 35,000 high-quality reference genomes stored in the *microbetagDB*. All annotations present in *microbetagDB* are also available through an application programming interface (API). *microbetag*’s source code is distributed under a GNU GPL v3 license and available on GitHub (https://github.com/msysbio/microbetag). Documentation and further support on how to use *microbetag* is available through a ReadTheDocs documentation page (https://microbetag.readthedocs.io/en/latest). To the best of our knowledge, there is no software with which *microbetag* could be compared to directly. To validate our annotations, we used a recently published network with partially known interactions [33]. We then present the main features of *microbetag*’s interface and discuss two extra application examples.

## Results

### Overview of the *microbetag* workflow and software ecosystem

*microbetag* integrates four sources of annotations—two at the node level and two at the edge level. It can operate in two modes: (a) on-the-fly, where user-provided taxa are mapped to their closest Genome Taxonomy Database (GTDB) representative genomes for downstream annotation; or (b) locally, using custom genome inputs. At the node level, phenotypic traits are assigned based on either the genome associated with each taxon (see “Genome-based node annotation” in Methods) or their taxonomic identity, using the curated literature-based FAPROTAX database (see “Literature-based node annotation” in Methods). At the edge level, *microbetag* infers potential metabolic interactions via two complementary approaches: (a) pathway complementarity and (b) seed complementarity (see “Pathway complementarity” and “Seed scores and complementarity” in Methods). The annotated network can then be visualized on Cytoscape thanks to a dedicated Cytoscape app (*MGG*). For a complete overview of the workflow, refer to “The microbetag workflow” section in Methods.

The *microbetag* software ecosystem consists of five main modules: (a) *microbetagDB* consisting of the precalculations for 34,608 reference genomes and their pairwise combinations (see Methods), (b) a web server hosting *microbetagDB* and the *microbetag* app to annotate a co-occurrence network on-the-fly or to retrieve annotations through an API, (c) a Cytoscape app called *MGG* that enables users to easily invoke the workflow and investigate the annotated network, (d) a preprocessing workflow for data sets with more than 1000 sequence identifiers (OTUs/ASVs/bins, etc.), and (e) a stand-alone version of the *microbetag* precalculation steps combined with the *microbetag* workflow that supports annotating a network based on the user’s reference genomes.

**Fig. 1:**
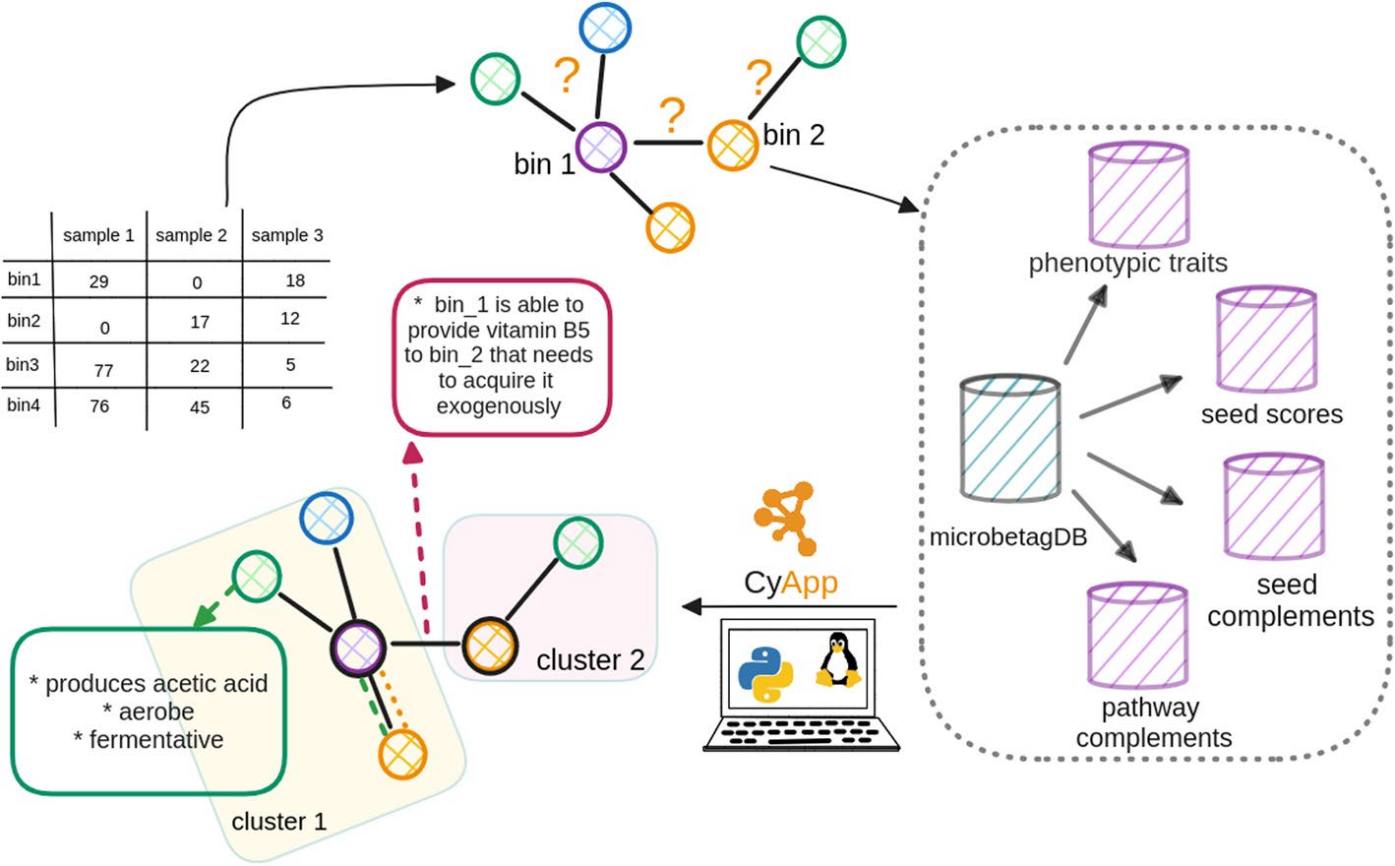
Simplified overview of the *microbetagDB*-dependent version of *microbetag* using an illustrative abundance table and its bins. From abundance data, microbial co-occurrence networks can be built (node colors represent different bins). Such networks consist of associations (edges) that are usually not directed and do not provide any interaction mechanisms, among other limitations. To tackle the challenge of interpreting microbial co-occurrence networks, *microbetag* adds a series of annotations at the node and edge levels. When used on-the-fly, *microbetag* makes use of *microbetagDB*, which stores precalculated phenotypic traits and metabolic complementarities among all possible pairwise combinations of high-quality, representative GTDB genomes. We have also developed a Cytoscape app called MGG to enable a user-friendly visualization of the *microbetag* derived annotations. Combined with a network clustering algorithm, *microbetag* also supports enrichment analysis of these traits within clusters. Node colors in the toy networks stand for different genera assigned to the sequences.

Figure 1 shows a simplified overview of *microbetag*’s on-the-fly annotation approach. Through the *MGG* Cytoscape app, the user may load an abundance table, and optionally its corresponding network, and send a job to the *microbetag* server. If not provided, *microbetag* will then infer a network using FlashWeave, and it will annotate its nodes (i.e., taxa) with phenotypic traits and its edges (i.e., significant co-occurrences or mutual exclusions) with potential metabolic complementarities. The annotated network is then returned to the user as a response to their job-query and is automatically loaded into Cytoscape’s main window. The user can then investigate its annotations further thanks to the *MGG* features (see “Annotating networks with microbetag” section).

For thorough instructions on how to use *MGG* and *microbetag*, answers to frequently asked questions and hints to address the idiosyncrasies of various data sets, the reader may visit the documentation web site (https://microbetag.readthedocs.io). For more information on how *microbetag*-annotated networks can be viewed through the *MGG* Cytoscape app, see also *microbetag*’s ReadTheDocs. Finally, *microbetag*’s Matrix community (https://matrix.to/microbetagcommunity:matrix.org) offers a dedicated communication platform where users can directly report issues, ask questions, exchange feedback, and suggest new features.

### The *microbetagDB* resource

Currently, *microbetagDB* includes more than 34,000 genomes (Table 1) along with their corresponding annotations. Most of these genomes represent bacterial taxa (364 taxa are archaea). The presence/absence of more than 30 phenotypic traits have been predicted for those genomes; in Additional file 2: Table S1, we list those traits along with their corresponding abbreviations on the MGG Cytoscape app. About 1.4 billion potential metabolic interactions leading to pathway or seed complementarities have been precomputed as well. In the *microbetag* framework, *pathway complementarity* occurs when, based on their genomes, a species (donor) provides missing enzymes or pathways to another species (beneficiary), enabling the beneficiary to complete a KEGG module (see “Pathway complementarity” in “Methods”). A total of 341,568 unique pathway complementarities, i.e., sets of KEGG terms supporting the completion of a module, gave rise to the 184.2 million pairwise pathway complementarities observed. Similarly, *seed complementarity*, following the definition in Lam et al. [27], emerges when a beneficiary’s seed metabolite(s) are produced by the donor’s metabolic network, enabling potential cross-feeding. Using the sets of the metabolites a species can produce on its own (non-seed set) and of its seeds, cooperation/competition scores were calculated (see “Seed scores and complementarity” in “Methods”). Currently, *microbetagDB* contains seed and non-seed sets for 33,755 GTDB representative genomes, which are being used to compute seed complementarities for any pairwise combination of these genomes on-the-fly. All annotations can be accessed directly from *microbetagDB* through an API.

**Table 1:** Summary of the data in microbetagDB

Description	Entries
GTDB representative genomes	34,608
Phen-model-oriented metabolic functions	32
FAPROTAX functions	92
Metabolic networks	33,755
Unique pathway complements	341,568
Pairwise pathway complementarities	184,184,548
(non-)seed sets	33,755

### Annotating networks with *microbetag*

The *microbetag* annotation pipeline can be performed either locally using custom genomes or on-the-fly, after mapping taxa to their closest GTDB genomes. In both cases, the annotated network will be viewed in Cytoscape through the *MGG* Cytoscape app. The *MGG* app is also the one supporting running *microbetag* on-the-fly, by importing data in the app and submitting a job to *microbetag*’s web server.

More specifically, *MGG* was developed to simplify the use of *microbetag*; thus, running *microbetag* on-the-fly is straightforward and requires no bioinformatics skills. In the simplest case, *microbetag* only requires an abundance table with taxonomic assignments as input. When the user-provided taxonomy scheme is not one among the GTDB, Silva, or the GTDB-oriented taxonomy for 16S rRNA amplicon data (see “*microbetag* preprocessing”), *microbetag* maps the user’s taxonomy to an NCBI Taxonomy Id and from that to GTDB representative genomes; this mapping is the most time-consuming step of the workflow. Network inference can be a computationally intensive step too, particularly as the number of taxa in the abundance table increases. To enable annotation of large data sets, a stand-alone preprocessing workflow is provided with *microbetag*. The user can either assign their amplicon data to the GTDB-oriented taxonomy and/or reconstruct a network locally. Once a network is available and the taxonomy provided is among the standard ones for *microbetag*, the computational time required for annotation ranges from several seconds to just a few minutes based on the user’s settings. An annotated network in .cx2 format is then returned, which can be viewed in Cytoscape.

To enable the *microbetag* approach with custom/local genomes, a *microbetagDB*-free version is also available. In this case, the user needs to run the *microbetag* pipeline locally, either installing *microbetag* and its dependencies from source or as a container. *microbetag* will first perform all the genome annotation steps and the metabolic model reconstruction if needed, depending on the user’s settings. For example, in case a user has already annotated their genomes with KEGG, or they have already reconstructed their corresponding metabolic networks, these precalculation steps may be omitted. Then, the *microbetag* workflow (see “Methods”) will be performed to return an annotated network. The computing resources for such a task can be quite extensive (see “Run times”). Once completed, the annotated network will be saved as a .cx2 file and can be visualized in Cytoscape using the MGG app.

**Fig. 2:**
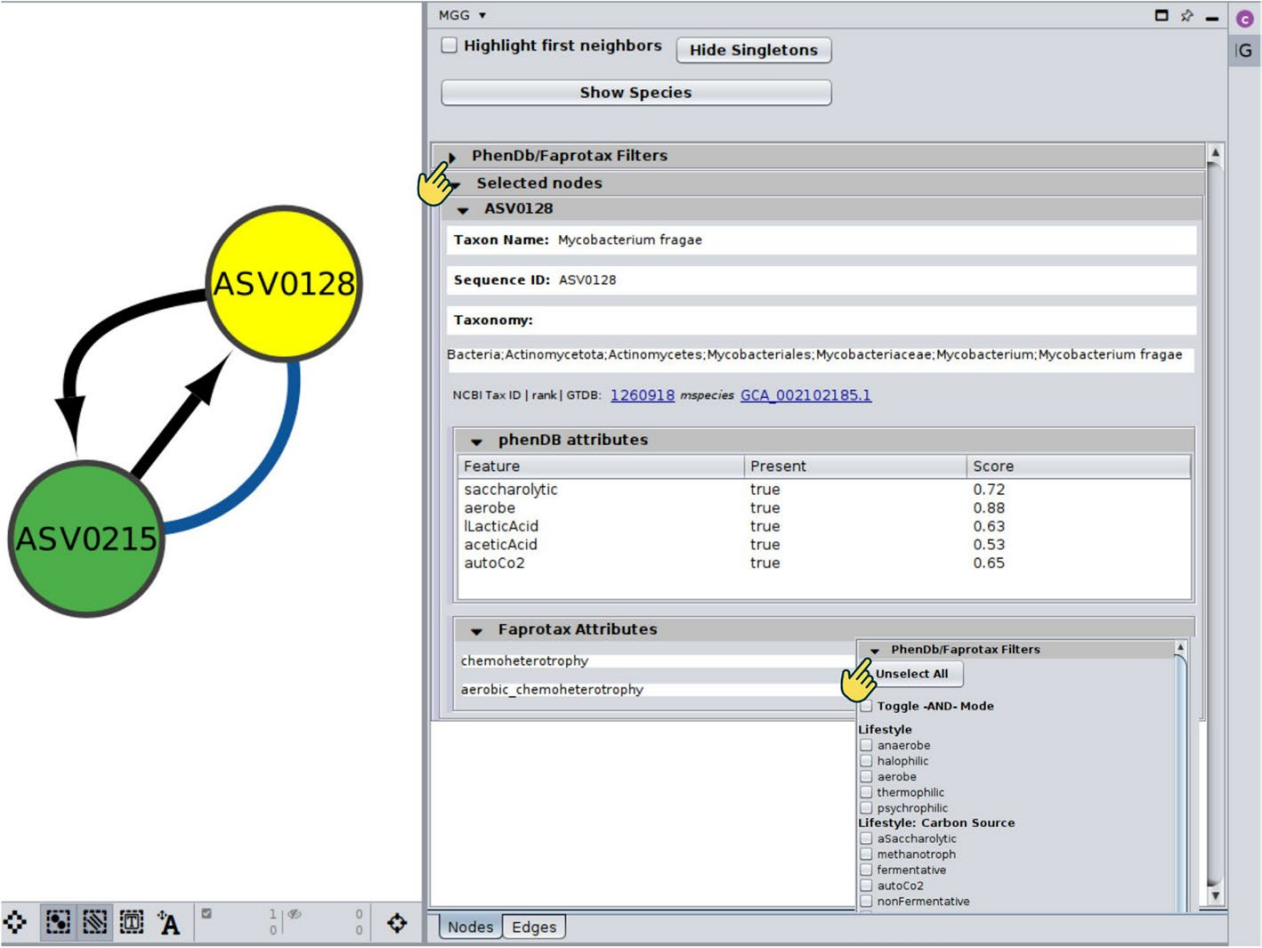
*Nodes* CyPanel of the *MGG* Cytoscape app. Once a node is selected, the *Nodes* panel displays its taxonomy, the NCBI Taxonomy id to which it was mapped, and its corresponding taxonomic level, and if available, the GTDB genome(s) to which it was mapped. In this example, an ASV that was assigned as *Mycobacterium fragae* was mapped to the GCA_002102185.1 GTDB representative genome, based on which a set of phenotypic traits was predicted to be present along with a prediction score. Also, based on FAPROTAX literature-based annotations, the ASV was annotated as chemoheterotroph. When multiple nodes are selected, a collapsible panel—similar to the one shown for ASV0128 in this example—will appear. On top of the panel, there is a “control area” with a set of features that allow the user to filter the nodes to be displayed. The “Show species” button filters taxa so that only those that have been matched to a genome are shown in the main panel, while the “Highlight first neighbors” highlights all nodes that are directly connected with a selected node. After clicking on the “PhenDB/Faprotax Filters” collapsible panel, the complete list of phenotypic traits across all taxa in the network is shown, organized in groups (see “Groups of phenotypic traits” in “Methods” for more). From there, the user may select to show only nodes (taxa) carrying either at least one (OR) or all (AND) of a set of traits.

*MGG* allows the user to import data, retrieve an annotated network, and investigate the annotations through a series of CyPanels both for node and edge annotations. Figure 2 shows an example of the *Nodes* CyPanel. The node name, taxonomy, NCBI Taxonomy Id, and GTDB genome to which the sequence was mapped (in case of an on-the-fly analysis) can be viewed. Depending on the user’s settings and the available annotations for a node, genome- or literature-based predictions may be presented. Further, the trait groups mentioned in “Groups of phenotypic traits” are displayed on top of this panel allowing for the selection of the nodes carrying either one among several attributes (OR logical relationship) or all of them (AND) (Fig. 2).

**Fig. 3:**
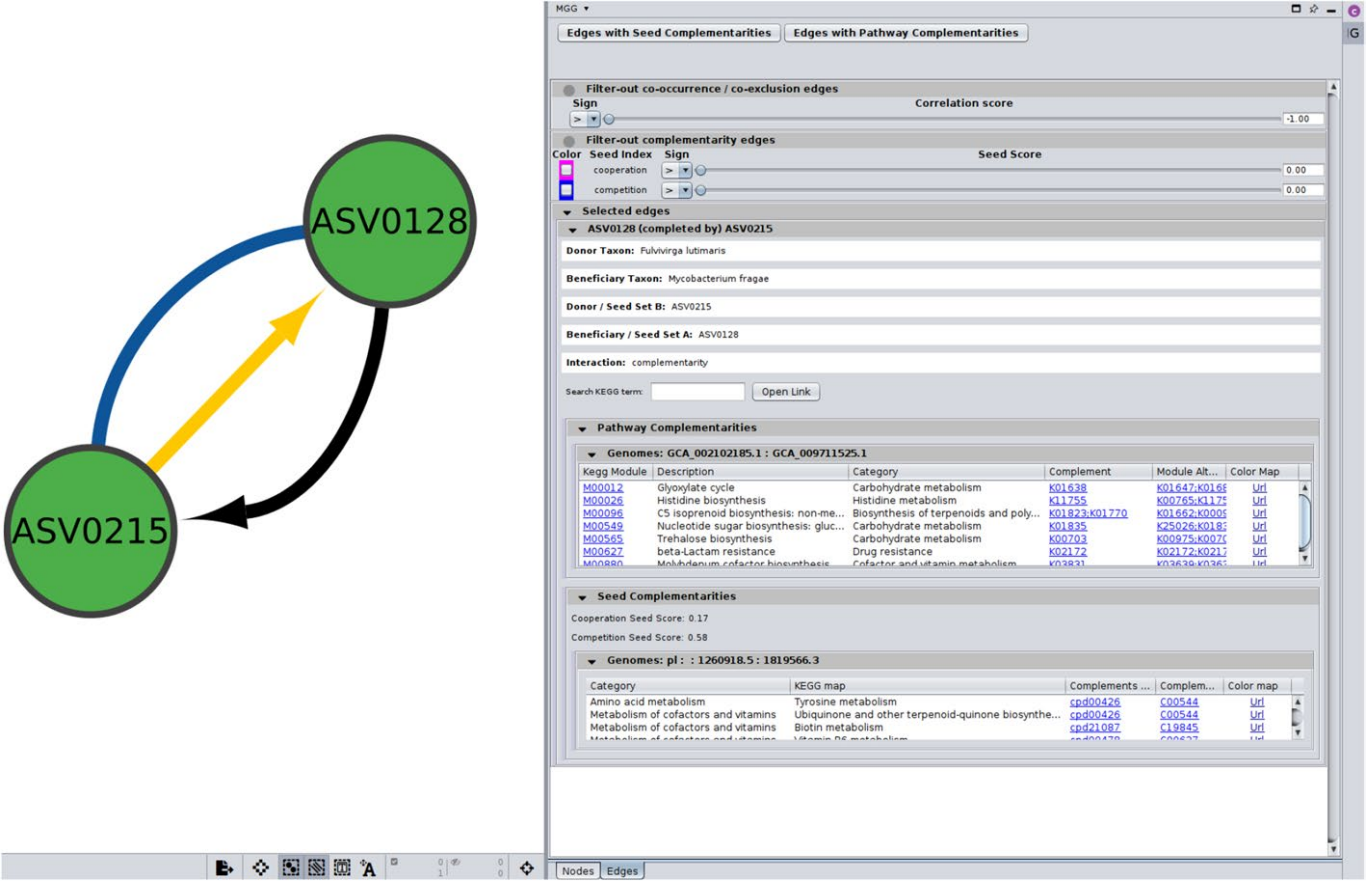
*Edges* CyPanel of the MGG Cytoscape app. Once an edge is selected, the edge panel displays the names of the two taxa involved and their corresponding sequence ids, as well as the interaction type. In case of a complementarity edge, a sub-panel for each complementarity type is provided. For pathway complementarities, the KEGG module being complemented is shown in the first column, followed by its description and the metabolic category it belongs to. The corresponding KOs related to the suggested cross-feeding compounds are also listed, along with the completed version of the module based on these additions. A URL is provided linking to a color-coded KEGG map that highlights the module under study (Fig. 4A). Likewise, for seed complementarities, a collapsible table is provided showing the category type and the associated KEGG map to which the seed compound contributes. It includes the seeds that can potentially be cross-fed from the donor, listed in both ModelSEED and KEGG namespaces. A URL is provided linking to a color-coded KEGG map highlighting both the beneficiary’s related compounds and the potential seeds it could acquire (Fig. 4B). Seeds linked to multiple KEGG maps may appear repeatedly in the table (see C00544). As in the *Nodes* panel, edges are filtered via the top control area. They can be either of “co-occurrence” type (undirected, unless using a custom network inference method) or “complementarity” (directed, with the source as the donor and the target as the beneficiary). The “Edges with Seed Complementarities” and “Edges with Pathway Complementarities” hide any edges that do not carry such annotations. The sliders below filter edges based on a score, allowing the user to set a threshold that can include values either above or below it. Slidebars can be combined.

**Fig. 4:**
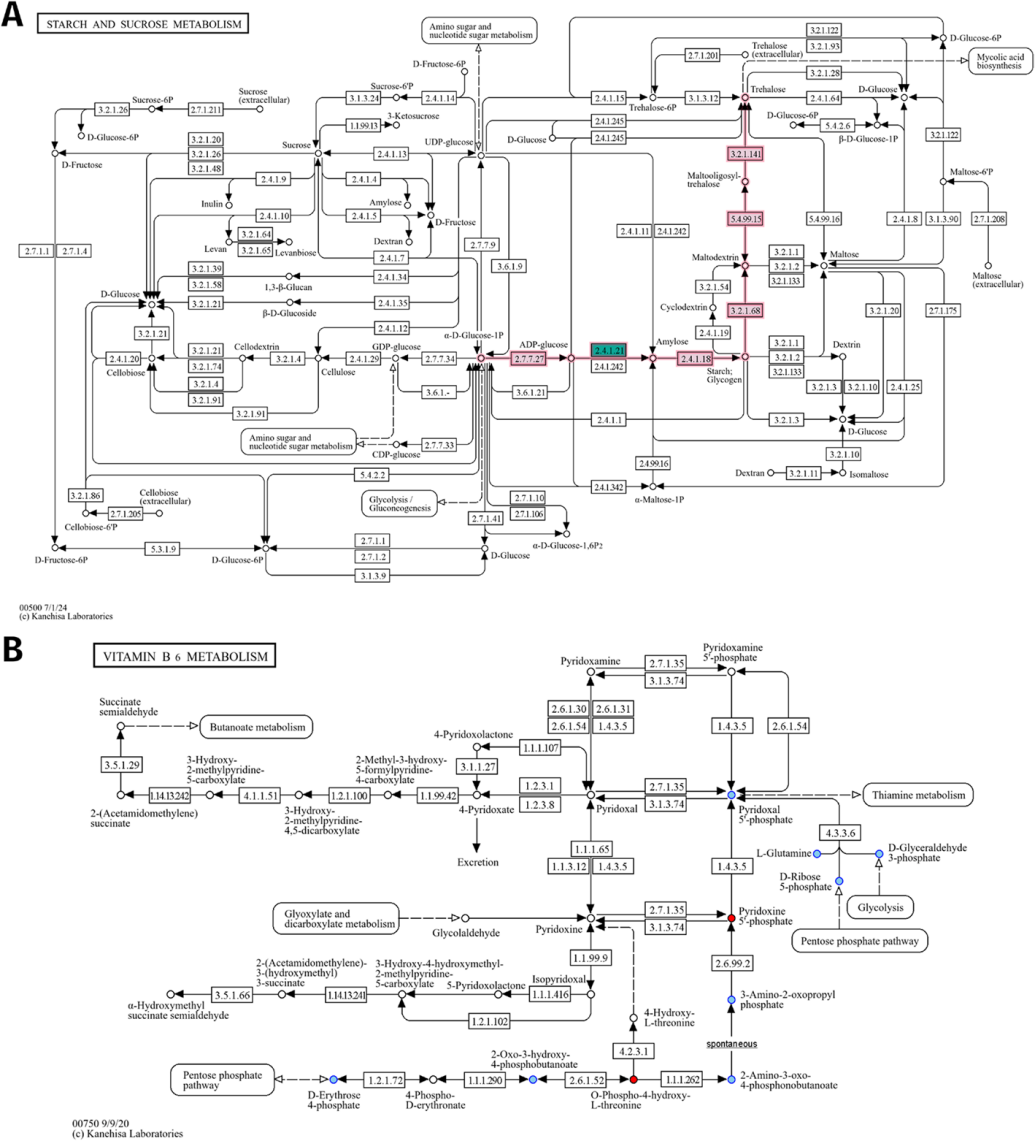
Colored KEGG maps illustrating the suggested completed mechanism and highlighting the impact of the corresponding potential metabolic interaction with the overall species metabolism. *microbetag* constructs URLs using the KEGG API to visualize the complementarities found between two taxa. **A** In case of “pathway complementarity,” a set of missing KOs is provided to complete a specific KEGG module. In this example, module M00565 (trehalose biosynthesis) would be complete after acquiring a starch synthase, represented by K00703 (EC:2.4.1.21). This approach is unaware of alternative ways for the species to achieve the same task. For example, in this case, we do not know whether the species could use another pathway to synthesize trehalose. **B** In contrast, “seed complementarity” identifies as potential cross-fed metabolites those compounds that the beneficiary cannot produce on its own by any means. The returned complements may contribute to multiple parts of the species’ metabolism. In this example, the strain donor could potentially provide the beneficiary with two seed compounds: 4-phosphonooxy-L-threonine (C06055) and pyridoxol 5′-phosphate (C00627)

Similarly, in the *Edges* panel (Fig. 3), the set of beneficiary taxa is specified, along with their corresponding GTDB representative sequence identifiers, while pathway and seed complementarities are listed in collapsible tables. Potential metabolic interactions are shown in a sub-table, bearing as title the genome pair under consideration as several GTDB genomes may have been assigned to a node. In case of pathway complementarities, these tables consist of six columns: (a) the KEGG MODULE id of the module to be completed, (b) its description, (c) a more general metabolic category to which the module belongs, (d) the complement itself as a list of KEGG terms, (e) the alternative that now represents a complete module in the beneficiary, and (f) a URL that points to a colored KEGG map highlighting the complement (see “Pathway complementarity” in “Methods”). If clicked, the user’s default browser pops up displaying a colored KEGG map as shown in an example in Fig. 4A. Similarly, in case of seed complementarities, a nested sub-table is again provided, but this time having as a header of each sub-table their corresponding PATRIC ids, which were used to reconstruct metabolic networks (see “Seed scores and complementarity” in “Methods”). In this case, the KEGG metabolism category is mentioned for each complement and the potential compounds being cross-fed are shown in red, while metabolites being produced by the beneficiary species are shown in blue (Fig. 4B).

Last, *MGG* allows testing for enrichment or depletion of the phenotypic traits assigned to the nodes in each of the network clusters. Clusters are optionally assigned by manta [15] while performing the *microbetag* workflow or assigned by users either manually or with another network clustering algorithm (see use case on subgingival plaque microbiome data).

In the following two sections, we present a test case and two use cases, highlighting our approach’s potential. For a thorough description of *MGG*’s panels, features, and visual style, readers are referred to the corresponding page in *microbetag*’s online documentation.

### Test case: complementarities predicted by *microbetag* for a network with known interactions

We tested *microbetag* using the correlation network of Hessler et al. [33], which describes mine tailing-derived (materials remaining after extracting the valuable fraction from an ore) consortia cultivated in bioreactors under varying conditions. In this study, *Variovorax*, a thiamine producer, and its co-occurrence with a series of thiamine auxotrophs are discussed. The authors tested network predictions by performing co-culture experiments measuring thiamine production. Sequence bins corresponding to network nodes and the original network were obtained from the authors (personal communication). Using GTDB-tk [34], GTDB taxonomies were assigned to the bins and added to the original network, which was then annotated with the on-the-fly version of *microbetag*. In addition, the local version of *microbetag* was also executed for bin_55 (*Variovorax*) and its first neighbors, as they were found in the original co-occurrence network from Hessler et al. Both annotated networks are available on *microbetag*’s GitHub repository.

GTDB-tk returned GCA_001899795.1 as the genome closest to bin_55, assigning it to *Variovorax* sp001899795; in total, 11 of the 16 taxa involved were mapped to a GTDB representative genome (Additional file 1: Fig. S1). With respect to *Variovorax*’s predicted traits, *microbetag* suggested that this specific genome corresponds to an aerobe [35] that can grow autotrophically [36] and consume D-glucose, while producing ethanol and L-lactic acid [37]. Last, the type VI secretion system was suggested to be encoded on its genome [38].

Hessler et al. argue that *Variovorax* is an important source of thiamine and can supply it to neighboring species that cannot produce it (auxotrophs). Indeed, *microbetag* suggested several thiamine-related potential seed complements among the potential metabolic interactions between *Variovorax* and its neighbors, both with the on-the-fly version and locally using the study’s bins (Table 2, Additional file 2: Tables S2 and S3, and Additional file 1: Fig. S2). However, using the study’s bins, *microbetag*’s local version not only returned cases of potential thiamine support for taxa that did not have a match in the on-the-fly version, but also matched three taxa that did not provide any such complementarity (Table 2). Interestingly, the two KEGG compounds that were found as potential seed complements with the on-the-fly version, C01081 and C20246, were also found by the local version. Out of the 15 neighboring taxa of *Variovorax*, 10 were marked as potential thiamine beneficiaries. Of these, 7 were also predicted to be thiamine auxotrophs by Hessler et al. The only taxon that could be matched between the two studies and was predicted to be a thiamine autotroph in the original study was similarly classified by *microbetag*. Four taxa could not be matched between the studies, while three were predicted as auxotrophs in the original study but showed no seed complementarity with *Variovorax*. Potential thiamine-related cross-feeding interactions were also found in some cases between the neighbors themselves (Additional file 2: Table S4).

**Table 2:**
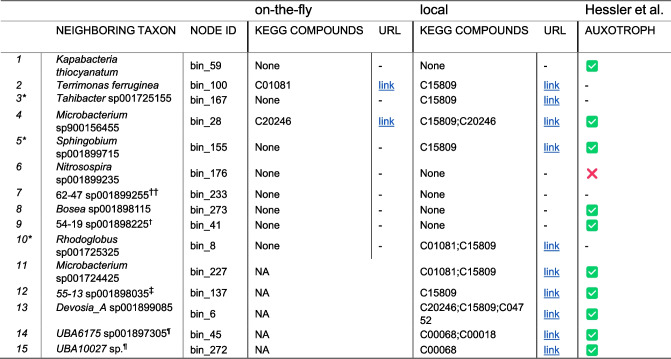
Thiamine biosynthesis related seed complements between *Variovorax* and its first neighboring taxa in the network of Hessler et al. [33] as returned by the on-the-fly version of *microbetag* and the GTDB taxonomy, and the local version of *microbetag* and the study’s genomes. The compound sets in the table rows are composed of only six compounds, namely C15809: iminoglycine; C01081: thiamin monophosphate; C04327: 4-methyl-5-(2-phospho-oxyethyl) thiazole; C20246: 2-[(2R,5Z)−2-carboxy-4-methylthiazol-5(2H)-ylidene]ethyl phosphate (cThz*-P); C00068: thiamin diphosphate; C00018: pyridoxal phosphate. Families of GTDB-specific taxonomy ids: ^††^Xanthobacteraceae; ^†^Chloroflexi; ^‡^Fimbriimonadaceae; ^¶^Candidatus Saccharibacteria.

The authors also argue that isolates of that particular *Variovorax* strain required the addition of pantothenic acid to grow. Based on the KEGG annotation of the genome that bin 55 was mapped to, it contains both KEGG modules related to pantothenate biosynthesis, M00119 (valine/L-aspartate ⇒ pantothenate) and M00913 (2-oxoisovalerate/spermine ⇒ pantothenate), whereas other genomes are not capable of either one or any of those reactions (Additional file 2: Table S5). Moreover, based on *microbetag*’s analysis of the bins from the Hessler et al. study, bin_55 was annotated with KOs K01918, K00606, and K00077—all of which are components of the “Pantothenate biosynthesis, 2-oxoisovalerate/spermine → pantothenate” module (M00913). Consequently, reaction R02473 was included in the metabolic reconstruction of *Variovorax* (bin_55), indicating that pantothenate was not among its seed metabolites. This highlights a key challenge of the *microbetag* approach—its qualitative nature may overlook the quantitative demand for compounds that are nonetheless essential. Nevertheless, several other vitamin B-related compounds were identified among the potential complementarities between bin_55 (as the beneficiary) and its neighboring donors, including biotin, riboflavin, and folate-related metabolites (Additional file 1: Figs. S3 and S4).

### Use case 1: cross-feeding may explain increased butyrate production in the presence of oligosaccharides

In their study, Cabrera et al. [39] showed that iron supplementation combined with either inulin or short-chain galacto-oligo oligosaccharides (scGOS) and long chain fructo-oligosaccharides (lcFOS) leads to a higher relative abundance of bifidobacteria, increased production of acetate, propionate, and butyrate, and a significant shift in infant gut microbial composition compared to non-supplemented microbiota. In this way, enteropathogens that are usually increased due to iron supplementation, a common approach to prevent anemia, were reduced.

**Fig. 5:**
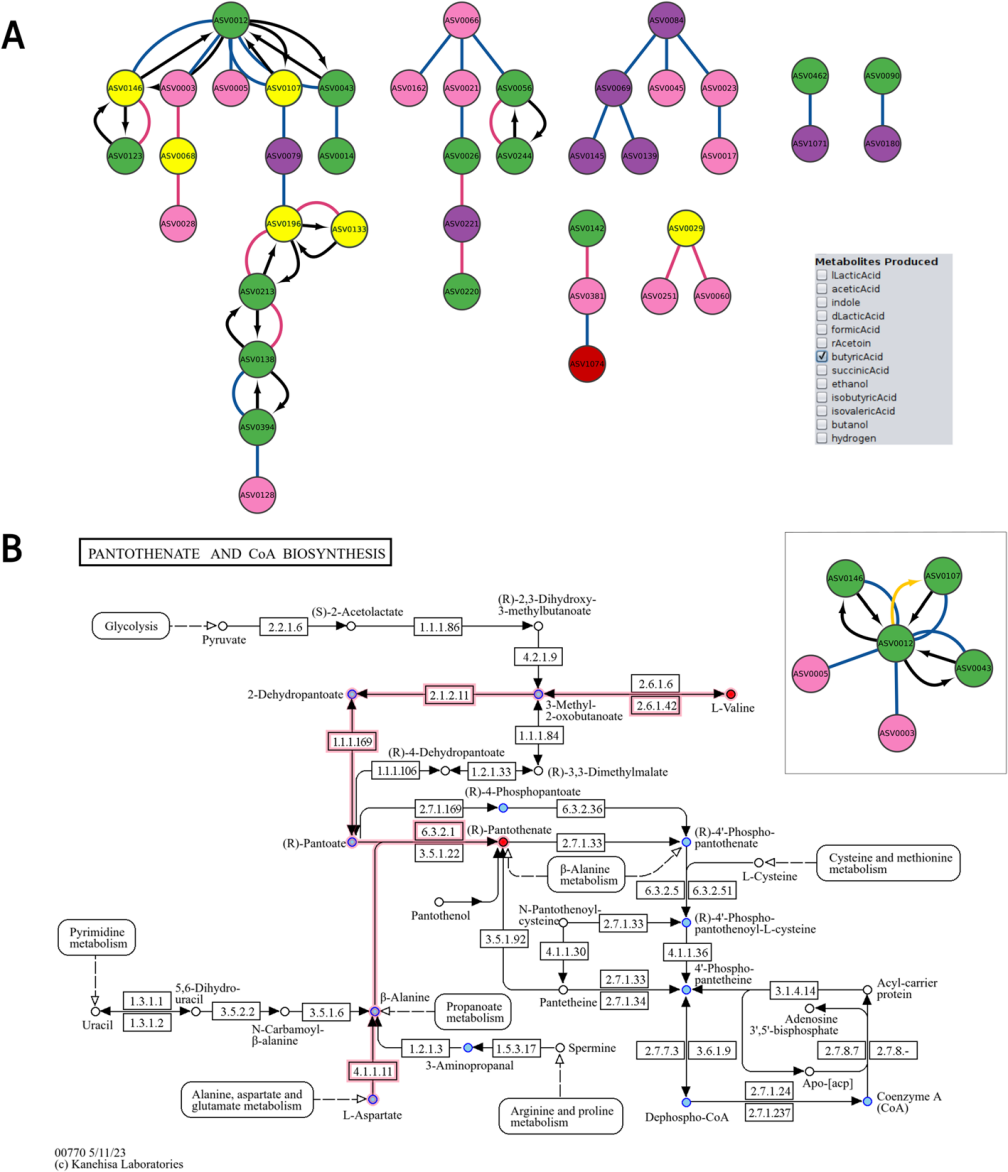
*microbetag* analysis of a dataset of infant gut microbiota studied *in* *vitro* [39]. **A** The *microbetag*-annotated co-occurrence network as viewed in Cytoscape with the MGG visual style. Taxa predicted to be butyrate producers are highlighted in yellow. Green nodes stand for taxa assigned at the strain or species level and mapped to a genome, thus carrying genome-based phenotypic predictions; pink and purple nodes stand for taxa assigned at the genus and higher taxonomic levels, respectively, and thus carrying only FAPROTAX-based annotations, if any. Blue and magenta edges stand for positive and negative co-occurrence scores, respectively, while black, directed edges were contributed by *microbetag* and represent pathway and/or seed complementarities with a donor taxon being the source and the potential beneficiary taxon being the target of the edge. Since both species in a pair can potentially act as donor and beneficiary, there are frequently two such directed edges connecting taxon pairs. *Flavonifractor plautii* (ASV0012) was positioned at the top of the far-left independent clade in the hierarchical layout. **B** Based on *microbetag*’s edge annotations considering *F. plautii* (ASV0012) as the potential beneficiary and *Eggerthella lenta* (ASV0107) as a potential donor, both pathway and seed complementarities are available (Additional file 1: Fig. S5), and complements related to pantothenate (vitamin B5) biosynthesis, and thus coenzyme A biosynthesis are present. The colored pantothenate and CoA biosynthesis KEGG map returned by *microbetag* highlights potential seed complementarities provided by *E. lenta*, L-Valine and (R)-pantothenate, in red. Based on the pathway complementarities, the same KEGG map suggests a missing enzyme (2.1.2.11) in *F. plautii* that could be acquired from *E. lenta* and that would enable *F. plautii* to produce pantothenate from pantoate (Additional file 1: Fig. S6). The selected edge turns orange; its annotations are displayed in the edge panel (as shown in Fig. 3).

To analyze these data further, we first ran the *microbetag* preparation step to map the ASVs in the 16S rRNA gene sequencing data of this study to GTDB-based taxonomic assignments. Then, we inferred the network with FlashWeave (wrapped by *microbetag*) and annotated it using *microbetag*. Figure 5 A shows the *microbetag*-annotated co-occurrence network for microbiota from donor 3, an 8-month-old female infant, whose fecal microbiota was used to inoculate the continuous *in vitro* experiment. After a stabilization period of 7 to 13 days, iron and scGOS/lcFOS were continuously supplied for 6 to 9 days, and this treatment set-up was repeated twice. *microbetag* predicted several taxa to be butyrate producers. Interestingly, most of the associations involving butyrate producers are negative, likely reflecting that they represent a different stage of the succession. At the same time, a single butyrate producer (*Flavonifractor plautii*; ASV0012) had several positive associations*. microbetag* reports that *F. plautii* and its neighbor species *Eggerthella lenta* (ASV0107) have several pathway and seed complementarities (Additional file 1: Fig. S5), in particular for coenzyme A biosynthesis, which is involved, among other tasks, in the bacterial butyrate production pathway [40]. More specifically, *F. plautii* requires pantothenate to be exogenously acquired, while *E. lenta* is capable of producing it. Thus, the analysis with *microbetag* results in a testable hypothesis: the observed increase in butyrate production and *Flavonifractor* in infant microbial communities upon FOS treatment *in* *vitro* (see Figs. 5B and 7 in [39]) may be, at least to some extent, due to cross-feeding partners boosted by the treatment that complement metabolic pathways involved in butyrate production (Fig. 5B, Additional file 1: Fig. S6). Relevant files for the use case and the *microbetag-*annotated network can be found at *microbetag*’s GitHub repository.

### Use case 2: enrichment analysis on *microbetag*’s node annotations suggests drivers behind clusters in subgingival plaque microbiome data

We recovered subgingival plaque amplicon data from the record of the Human Microbiome Project (HMP) [41] on Qiita [42] (ID 1928). The corresponding OTU table, consisting of 373 samples and 2057 taxa, was larger than the upper limit of 1000 taxa supported by the on-the-fly version of *microbetag*. Therefore, we first used *microbetag*’s preparation tool to assign GTDB-based taxonomies to OTUs and to infer a co-occurrence network with FlashWeave. With the output of *microbetag*’s preparation tool, we then annotated the network’s nodes with *microbetag* one step at a time. Then, we employed the *microbetag* stand-alone tool to cluster the network, which resulted in two clusters, with 504 (*cluster 0*) and 822 nodes (*cluster 1*), respectively. Among the nodes assigned to each taxon, 220 (44%) and 275 (33%) were mapped to a genome and thus were annotated with genome-based phenotypic traits.

We then performed an enrichment/depletion analysis and used the Benjamini–Hochberg method to control the false discovery rate (Additional file 2: Table S6), which is implemented in *MGG*. Cluster 0 was found to be enriched for the traits: acetic acid, aerobes, anaerobes, fermentative, L-lactic acid, and saccharolytic, while cluster 1 is enriched for D-lactic acid, human associated, human gut, hydrogen, and mammal gut. Our analysis also found the second cluster to be enriched with *Phocaeicola* and *Bacteroides*, i.e., taxa producing lactic and acetic acids, as well as with hydrogen producers. The two clusters appear to represent distinct states of the subgingival plaque microbiome, with cluster 0 aligning more closely with a balanced, commensal profile, and cluster 1 reflecting a more dysbiotic state. This is further supported by the extensive presence of *Veillonella* representatives in cluster 1 as well as by *Porphyromonas gingivalis*; although *Porphyromonas* strains are present in both clusters, only cluster 1 contains a known pathogenic strain [43]. In addition, out of the 23 *Streptococcus* nodes in the first cluster and 39 in the second, only seven (three and four, respectively) were taxonomically assigned at the species level and therefore annotated by *microbetag*. Therefore, the impact of *Streptococcus* on the formation of this microenvironment cannot be fully assessed from the annotations. It is worth mentioning that we were able to make these observations with no preprocessing of the data and without metadata. Combining study’s metadata with network clustering and annotation would bring further insight.

### Complementarity statistics

**Fig. 6:**
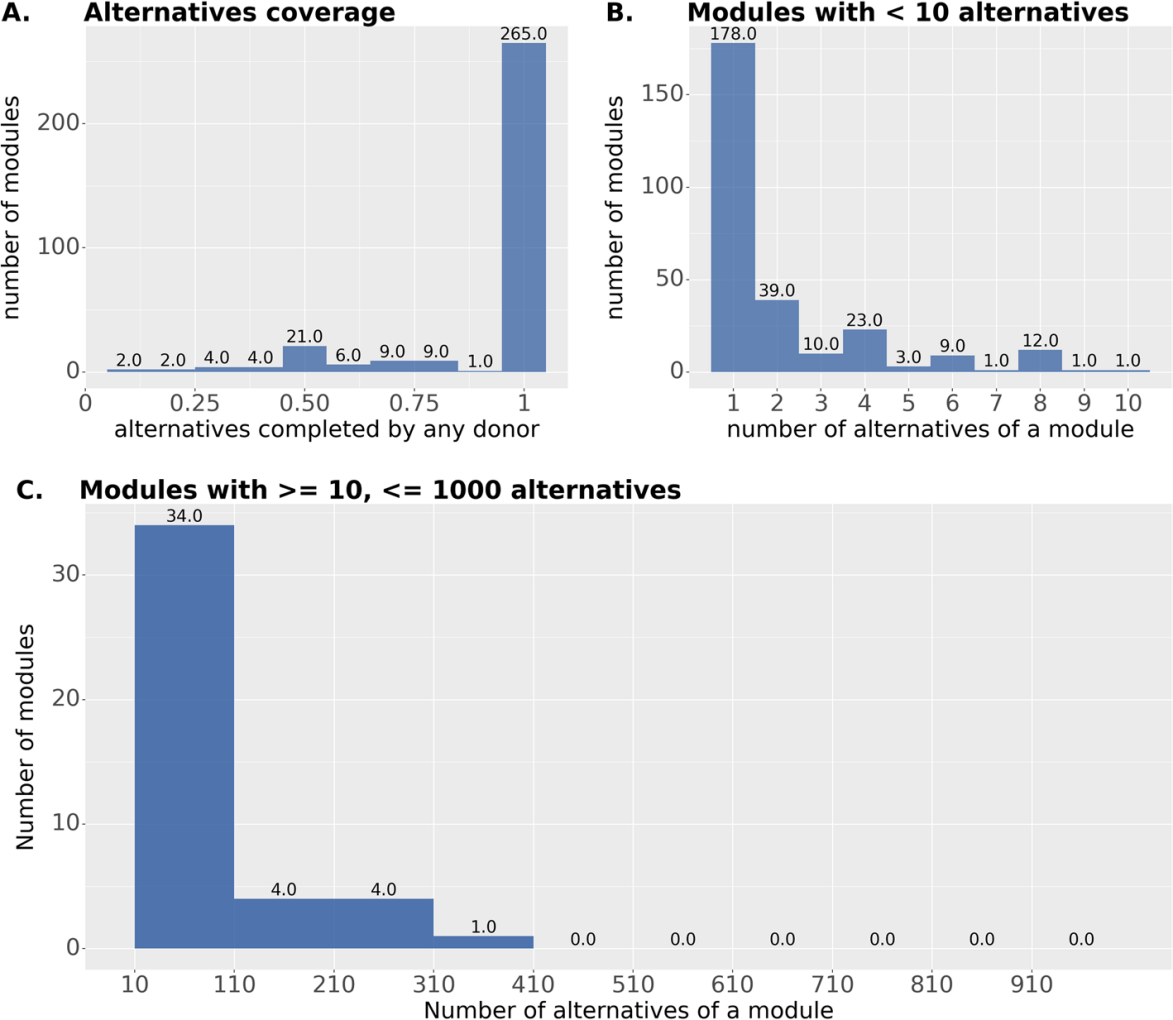
Statistics on alternatives for the KEGG modules available in *microbetagDB*. An *alternative* is defined here as a sub-network of a KEGG module that ascertains that the end products of the module can be produced. **A** For the vast majority of the modules, all their alternatives can be completed by a potential donor species (265). Overall, 323 out of 491 KEGG modules could be completed through at least one specific configuration of alternatives. **B** and **C** Most of the KEGG modules have 1 or 2 alternatives but there is a set of 132 modules for which the number of alternatives ranges from 3 to 13,440 (Embden-Meyerhof pathway (glycolysis), M00001).

In its current version (v1.0.4), *microbetag* supports complementarities for 491 KEGG modules. These modules have been analyzed to identify 23,592 unique *alternatives*, each of which is a sub-network of the module which ascertains that its end products can be produced. Using the.faa files of the 16,902 high-quality GTDB genomes, a total of 184.2 M pairwise pathway complementarities were retrieved (where a complementarity or complement is a combination of KOs that complete an alternative and thus enable a KEGG module). Under the “Pathway complementarity” section in “Methods”, a thorough description of the genomes considered and the pipeline to retrieve such complements is provided. Theoretically, one would expect 285.5 M pairwise complementarities, yet not every possible pair of genomes has a complement. More specifically, 237,075 unique complements were found to complete 6467 alternatives in a total of 341,568 unique pathway complementarities. The latter is due to the fact that the same complement may complete more than one module at the same time. It is worth mentioning that almost 15% of those are related to glycolysis; this is expected since 13,440 of the alternatives are related to this module. However, for the vast majority of the modules, all the enumerated alternatives were found to be potentially completed by another taxon (Fig. 6A).

In addition, 56 modules were found to carry more than 10 alternatives, covering 96% of the alternatives observed (Fig. 6B, C). Yet, 49,944 of the total 341,568 unique complementarities come from the remaining 435 modules. We also filtered the unique complements for those requiring more than 4 KOs from the donor species; we assume that the more KO terms need to be shared, the more challenging it is for the complementarity to occur. We found that out of the 215,883 cases requiring more than 4 KOs, 185,937 were found among the 56 modules with the highest number of alternatives.

The KEGG modules’ definitions cover all domains of life. However, some *KO terms* are only present in animals or plants, etc. This explains partially the alternatives that are never completed. For example, in the case of md:M00002, the term K12406 is only present in animals. Thus, from the total 24 alternatives for that module, only 12 are present in microbetagDB, as expected. Similarly, there are also *KO modules* that can occur only in plants and/or eukaryotes. This explains to a great extent why for 168 modules, no alternatives were completed at all. For example, module M00372, the “Abscisic acid biosynthesis,” is not present in *microbetagDB* as expected. There are also 3 KEGG modules with more than 1000 alternatives.

**Fig. 7:**
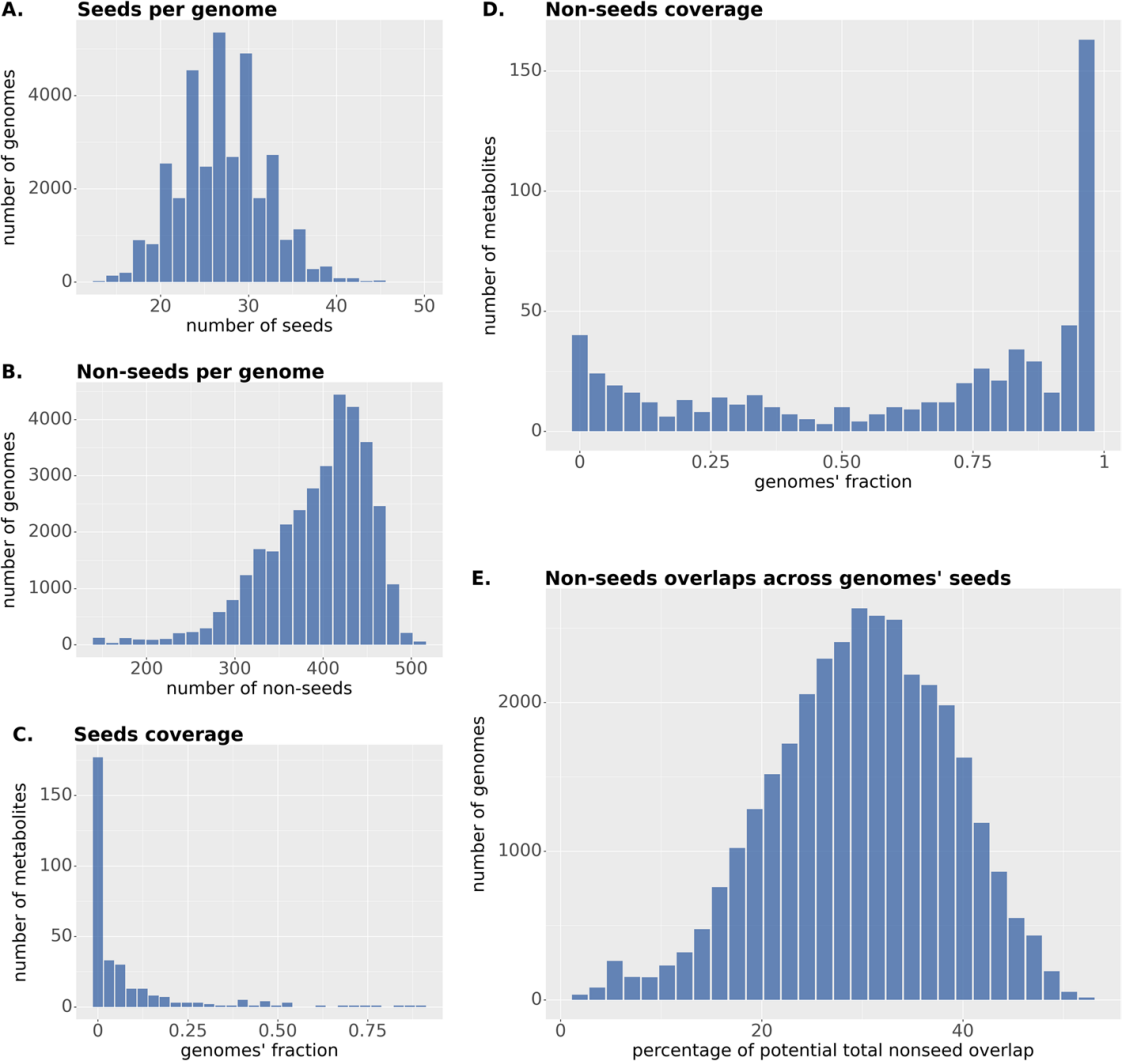
Distributions of seed and non-seed metabolites, including only those that are part of at least one KEGG module, across the ModelSEED reconstructions in *microbetagDB* v1.0.1, as well as of their overlap. **A** The number of seeds per genome is a symmetric distribution ranging from 13 to 51 seeds. **B** The number of non-seed compounds per genome has a left-skewed distribution. **C** and **D** Distributions of the relative frequency of a metabolite being a seed or non-seed, with the seed distribution decaying quickly from 0 and the non-seed distribution decaying towards 1. **E** Normal distribution of the percentage of the non-seeds of a genome found to overlap with seeds of all the other *microbetagDB* genomes.

With respect to seed complementarities, only terms linked to KEGG modules were considered, resulting in a total of 1432 ModelSEED compounds. Among them, 452 metabolites were identified as seeds in at least one genome, while 545 metabolites were found as non-seeds in at least one genome. As shown in Fig. 7A and B, in both cases there is a slightly right-skewed distribution, especially in the case of non-seed compounds, with an average of 99 seed compounds and 333 non-seeds. The majority of compounds are seeds in small sets of genomes with only 81 compounds being a seed in more than 50% of the genomes (Fig. 7C), while a total of 270 compounds could be potentially produced by more than 75% of the genomes (Fig. 7D).

Theoretically, if a genome’s non-seed compounds could be cross-fed to complete all seeds across the rest of the genomes, this would result in approximately 15.3 M overlaps. As shown in Fig. 7E, the percentage of potential cross-feedings follows a normal distribution, with an average of 3.2% of a genome’s non-seeds appearing as seeds in other genomes. This suggests that most genomes have a similar potential to provide seed compounds, with only 16 genomes exceeding this average by more than three standard deviations and just one genome falling below it by the same margin. Notably, this one genome represents *Ureaplasma diversum* NCTC 246, a facultative intracellular strain of the Mycoplasmataceae family, which includes several parasitic and saprotrophic taxa.

Table 3 highlights the most and least common metabolites found as part of the seed set of the *microbetagDB* genomes. The presence of essential compounds—such as ATP and phenylpyruvate—that no organism can survive without, as the least frequent seed compounds serves as a positive validation of our pipeline. L-aspartate may be among the least common seeds because cells typically make aspartate and glutamate from TCA intermediates using transamination (an amino group is transferred from an amino acid to a keto acid) and since transaminase annotation is often challenging, most metabolic network reconstruction tools assume their presence by default [44]. It is also not surprising that cThz-P and L-2-amino-6-oxo pimelate are among the most common seeds since a large number of taxa are auxotrophic for thiamine or thiazole intermediates, and/or lack parts of the diaminopimelate pathway. The other three compounds are part of pathways that are relatively rare among bacterial species; for instance, most bacteria do not degrade purines completely to allantoin, often utilizing alternative degradation routes instead.

**Table 3:** Most and least frequent seeds across the metabolic networks in *microbetagDB*. For the most frequent (TOP) seeds, we skipped the first four fatty acid metabolism-related compounds, which are artifacts of the ModelSEED reconstructions and are not being used except in rare cases

	Compound	ModelSEED ID	KEGG metabolism categories
TOP	2-(2-Carboxy-4-methylthiazol-5-yl)ethyl phosphate (cThz-P))	cpd21480	Thiamine biosynthesis
	Geranylgeranyl diphosphate	cpd00289	Ubiquinone and other terpenoid-quinone biosynthesis
	2-Oxo-4-hydroxy-4-carboxy-5-ureidoimidazoline	cpd09027	Purine degradation, xanthine = > urea
	L-2-amino-6-oxopimelate	cpd02414	Lysine biosynthesis, DAP dehydrogenase pathway, aspartate = > lysine
	5-Hydroxyisourate	cpd08625	Purine degradation, xanthine = > urea
Least	ATP	cpd00002	Amino acids biosynthesis
	Choline	cpd00098	Nucleotide metabolism
	Phenylpyruvate	cpd00143	Phenylalanine biosynthesis
	ADP-L-glycero-D-manno-heptose	cpd03831	Biosynthesis of nucleotide sugars
	L-aspartate	cpd00041	Carbon metabolism, biosynthesis of nucleotide sugars

### Run times

*Microbetag* offers various modules tailored to the unique characteristics of each dataset. In the following sections, we discuss run times for different scenarios to guide users in selecting the most suitable approach for their dataset. Run times range from seconds (s) to minutes (min) and hours (h). For an overview of *microbetag*’s workflow, see “The microbetag workflow” in the “Methods” section.

#### On-the-fly

Using the abundance table from the test case [33] (Table 4, *vitAbund.tsv*, 120 bins) annotated with the GTDB taxonomy scheme and its corresponding network file (*edgelist.tsv*, 102 nodes, 251 edges), it took 40 s for *microbetag* to return an annotated network; *microbetag* was able to annotate 55 of its nodes and 93 of its edges. Given a network, the computing time is independent of the number of samples. Next, using a different abundance table containing 16 samples and 78 Silva-annotated ASVs (testAbundMeta.tsv), *microbetag* took 67 s to infer a co-occurrence network with 23 nodes and 14 edges using the FlashWeave sensitive approach and annotated 7 of its nodes and 1 of its edges. We then ran the same analysis with an abundance table that had the same ASVs as in the previous example but with 84 samples (*testAbund.tsv*). This time, it took 74 s for *microbetag* to return a co-occurrence network of 78 nodes of which 19 were annotated and 118 edges of which 5 were annotated. Thus, as expected, the number of samples affects the time required to infer the co-occurrence network. To highlight the impact of the taxonomy scheme on the run time, we provided the returned co-occurrence network from this last case as input, setting the taxonomy scheme to “Other” (instead of “Silva” as selected initially). *microbetag* returned an annotated network with only 16 annotated nodes and 3 annotated edges, while it took an extra time of 52 s to map the taxonomies against NCBI. Overall, since the on-the-fly module is capable of running jobs that require no more than a few minutes, it is recommended for cases involving small datasets or for scenarios where a co-occurrence network already exists and the taxonomy scheme is either Silva or GTDB.

**Table 4:** Run times of the on-the-fly and the preprocessing *microbetag* modules

	**Abundance table**	**Number of sequences**	**Number of samples**	**Taxonomy scheme**	**Network available**	**Run time**	**Number of nodes**	**Number of annotated nodes**	**Number of edges**	**Number of annotated edges**	**Notes**
On-the-fly	vitAbund.tsv	120	Invariant	GTDB	edgelist.tsv	40 s	102	55	251	93	
	testAbundMeta.tsv	78	16	Silva	NA	67 s	23	7	14	1	metadata.tsv
	testAbund.csv	78	84	Silva	NA	74 s	78	19	118	5	Same ASVs as in the row above (testAbundMeta.tsv)
		78	84	Other	edgelist.tsv	52 s	78	16	118	3	Network from the row above
Preprocessing	seq_ab_tab.tsv	16	997	GTDB	NA	232 s + 94 s	240	105	219	49	
					As returned from previous row	(+ 40 s)					

#### Preprocessing tool

The *microbetag* preprocessing tool provides a GTDB annotated abundance table and its corresponding co-occurrence network, both tailored for seamless integration with the on-the-fly version, facilitating the analysis of larger datasets. We used an abundance file (*seq_ab_tab.tsv*) of 16 samples and 997 ASVs and annotated them with a GTDB-based taxonomy. We also performed the FlashWeave step using the sensitive approach. In total, using a personal computer of 15 CPUs, it took 3 min 52 s real time, while the user time was 24 min 19 s implying a degree of parallelization; on average 6 CPUs were used. When providing an abundance table with taxonomies only, it took 94 s to return a network of 240 nodes, 105 of which annotated, and 219 edges, 49 of which annotated. When we used both the GTDB annotated table and the network, it took 40 s; the 54 s difference was the time FlashWeave required to build the co-occurrence network on the fly (Table 4). In addition, locally, one can further exploit the parallelization potential of FlashWeave thanks to its Julia implementation; in our tests, we did not make use of it.

#### Stand-alone version

Real-world data may lead to large co-occurrence networks consisting of thousands of nodes and edges. The on-the-fly version of *microbetag* is not suitable for such large networks. For this reason, we developed a stand-alone version, which not only handles large networks better but also supports genome-scale metabolic reconstruction for custom genomes. Custom genomes require an important amount of computing resources, especially in two steps: gene prediction and KEGG annotation of the genomes. In case genome-scale reconstructions are carried out with ModelSEEDpy, then RAST annotation would also be required. For instance, in our demo case, with only 7 bins to annotate, it took 1 h for Open Reading Frame (ORF) detection (running prodigal through DiTing) and about 2 h for the KOs assignment using a personal computer with 2 CPUs. Reconstructing the metabolic models with *carveme* is faster and performs in a more robust way since there is no need for establishing a connection with external servers as in the ModelSEED case, where a connection with the RAST server is required. Overall, the stand-alone version supports large datasets, with computing times varying significantly depending on the user’s parameter settings.

## Discussion

### Potential and limitations

The previous paragraphs illustrate the potential of *microbetag* in the interpretation of co-occurrence networks and how it can be used to generate new hypotheses derived from those. However, *microbetag* benefits the microbiome community in several other ways. *microbetagDB* provides a vast number of annotations: 31 predicted traits for more than 30,000 genomes, their metabolic networks along with their corresponding seed sets, potential metabolic complementarities, and cooperation/competition scores. Such a resource may support a range of studies; from a more theoretical perspective regarding the distribution of the complements among taxonomic groups or how often a complement potentially appears, to applications such as eco-evolution studies and the investigation of interactions. For example, the “Pentose phosphate pathway” module (md:M00004) was the one with the most alternatives [40] that could be completed by another species. At the same time, “De novo purine biosynthesis, PRPP + glutamine = > IMP” (md:M00048) is the module with the lowest percentage of alternatives completed by any donor (0.06%). A closer look to the latter shows that as part of the definition of the module, there are KOs that can be found only in non-bacterial taxa; e.g., among all the bacterial KEGG genomes, K11787 (phosphoribosylamine–glycine ligase) is present only in *Defluviicoccus* sp. SSA4 while K01587 (phosphoribosylaminoimidazole carboxylase) is only present in *Rhodoplanes* sp. Z2-YC6860 and *Berkelbacteria* bacterium GW2011_GWE1_39_12). In addition, K11787 is responsible for three steps of the module in case of *Homo sapiens* [M00048], suggesting a significantly different way of implementing the same task/module compared to prokaryotic taxa.

For studies with a small number of sequence identifiers, the on-the-fly version of *microbetag* returned annotated networks in a couple of minutes while in cases where a network was also provided it only took a few seconds. The two most time-consuming steps were network inference and matching non-Silva, non-GTDB taxonomies to GTDB genomes. When microbetag’s preprocessing was used, and its results were provided as input, the computational time for the on-the-fly part was reduced to a couple of seconds even for larger networks. The computing requirements for running *microbetag* with custom genomes are strongly dependent on the number of bins/MAGs/genomes and whether annotation steps and GEM reconstruction have been already performed or not.

Yet, there are several challenges involved in our approach. First, microbetag inherits all the biases and drawbacks of both the data and the software it is based on. Functional annotation comes with its own limitations. Some functional domains boast richer annotations and more comprehensive descriptions compared to others, which may be partly due to processes being studied at different depths (e.g., glycolysis versus biosynthesis of secondary metabolites).

In the test case (data from [33]), the bin representing the *Variovorax* strain was mapped to a genome that is supposed to contain the pantothenate KEGG modules. Thus, the fact that it requires pantothenate to grow, as the authors mention, would not have been predicted in the microbetag framework. This highlights the importance of strain-level variety and also the difference between genomic potential versus expression and synthesis of enzymes. Beyond the sequencing and annotation challenges, we also need to consider the fact that a pathway may not be fully represented in a KEGG module or that an organism may have alternative pathways for its product(s).

Pathway complementarity can only be as accurate as the KEGG MODULE database and as precise as the software annotating genomes with KO terms. In addition, pathway complementarity per se does not guarantee that intracellular metabolites are indeed exchanged; *microbetag* does not check whether metabolites can be excreted or consumed. If the donor lacks a transporter and the metabolite cannot cross the cell wall, sharing could still occur through lysis, but that requires a sufficiently high death rate. With respect to seed complementarity, it is well-known that automated metabolic reconstruction comes with a number of challenges, and different tools for this task have their own limitations [45]. As a result, different reconstruction approaches can lead to different metabolic networks and thus, to different seed and non-seed sets of each species. In *microbetagDB*, seed complementarities have been precalculated using metabolic networks built with ModelSEED and a complete medium, which may limit potential metabolic interactions but the retrieved ones will be more reliable because using a complete medium to gapfill a metabolic network reduces the number of reactions that need to be added. In general, interaction prediction based on the seed approach does not take into account the environment. To some extent, this can be addressed when using the stand-alone version of *microbetag*, where the user can work with their own metabolic models or reconstruct them using specific in silico media. The fact that the environment is currently not considered also means that seeds are consistent across networks, since different seed sets would arise in different environments. Furthermore, we do not check whether any of the seed-derived metabolites is required for growth. Seeds not required in rich environments or only needed for non-essential compounds lead to false positives and thus an overestimation of potential cross-feeding interactions. These are all reasons why pathway and network complementarities are predictions of potential cross-feeding relationships and do not necessarily reflect actual interactions.

In addition, pairwise relationships do not capture higher-order ecological interactions, in which species depend on (or are influenced by) multiple other species [1]. However, since microbetag is decoupled from network inference, it could annotate a network with hyperedges (i.e., edges connecting more than two taxa) produced by a future tool capable of inferring higher-order interactions.

Last, the limited number of archaea in microbetagDB is also the result of a software limitation. As shown in ([46]; Fig. 6b), the original version of CheckM [47] that is still being used by GTDB returns lower completeness scores for genomes that correspond to phyla known for having smaller genomes in general, e.g., Patescibacteria representative genomes in GTDB have an average completeness of ∼65%. Thus, only a few representatives from these taxonomic groups passed our filters leading to an important under-representation of archaea.

### Context

*Microbetag* is among a number of tools that are based on the reverse ecology approach, whose goal is to derive ecological insight, in particular on interactions, from the genomes of community members [25, 48–50]. *microbetag* goes beyond these previous tools and methods by combining interaction prediction based on metabolic networks not only with microbial network inference but also with the systematic annotation of taxa with phenotypic properties. In addition, it makes all these approaches accessible to researchers without bioinformatics skills. As such, it is a unique resource of potential use to everyone working with microbiome data.

### Future work

In the near future, we plan to develop two main features: (a) the integration of transcriptomics data provided by the user, which would enhance or lower the probability for a potential metabolic interaction to occur based on whether the KO terms involved are present or not, and (b) the integration of spatial data; it is well-known that the distance between cells determines whether an interaction occurs [51]. For this, we intend to support data with spatial dimensions. We may also consider the integration of additional phenotype prediction tools, such as bacLIFE [52].

## Conclusions

Co-occurrence networks are widely used in microbiome studies to explore associations. However, their inference and their interpretation come with several challenges. Metabolic exchanges among microbial taxa are considered ubiquitous [53] in a large number of environments. In our study, we applied the reverse ecology paradigm [23, 24, 29, 54, 55] and publicly available genomic data and software to predict phenotypic traits and construct metabolic networks to annotate co-occurrence networks derived from amplicon or shotgun data. Our annotation was in agreement with the study of Hessler et al. [33] predicting thiamine-related metabolic interactions among *Variovorax* and its closest neighbors, suggesting several ways to achieve them. Using the Cabrera et al. dataset [39], we showcased the potential of *microbetag* as a hypothesis-generating tool suggesting mechanisms for the increase of butyrate producers in infant microbiota supplemented with iron and GOSFOS. *microbetag* is the first one-stop-shop platform for the annotation of microbial co-occurrence networks, highlighting the potential of data integration combined with follow-up analyses for network interpretation.

## Methods

### Genomes included

Using the GTDB v207 metadata files for bacteria and archaea, we retrieved the NCBI genome accessions of the high-quality representative genomes, i.e., completeness ≥ 95% and contamination ≤ 5%. A set of 34,608 genomes was obtained, representing 25,294 unique NCBI Taxonomy Ids, since there are cases where more than one GTDB species maps to the same NCBI Taxonomy id. All genomes were annotated with COGs (see “Genome-based node annotation”). For 16,900 genomes with available amino acid sequence files (.faa), these files were utilized to identify potential pathway complementarities between genome pairs (see “Pathway complementarity”). Additionally, when available, corresponding annotations from the PATRIC database [56] were retrieved to reconstruct metabolic networks (see “Seed scores and complementarity”).

### The microbetag workflow

**Fig. 8:**
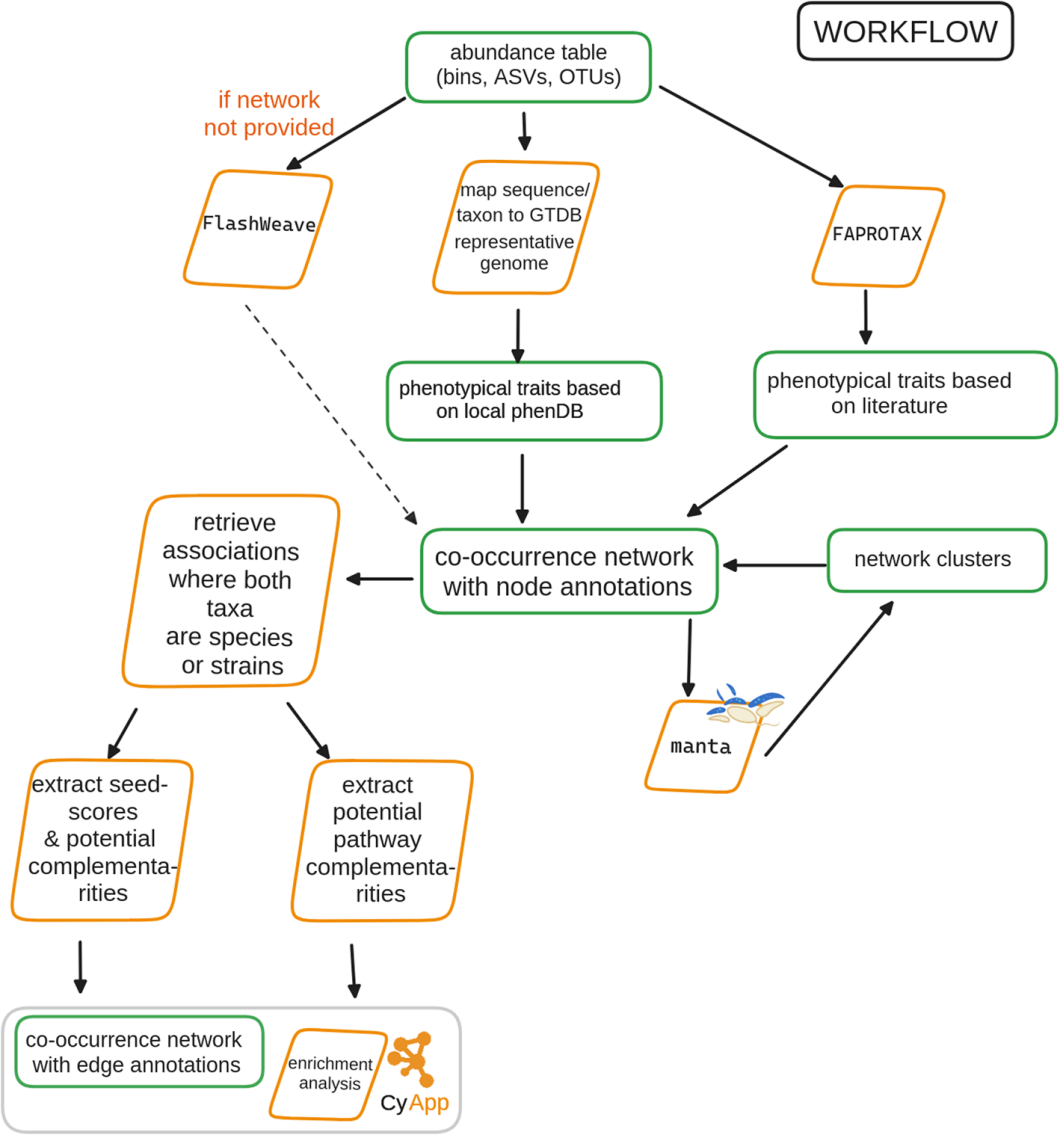
Diagram of *microbetag*’s on-the-fly workflow. *microbetag* expects either an abundance table as input and infers a microbial network using FlashWeave or an abundance table along with an already inferred network. After mapping taxa to GTDB reference genomes, for those with sufficient taxonomic resolution, phenotypic attributes are assigned to the nodes. Literature-based annotations of the nodes are also added using FAPROTAX. On the edge level, microbetag assigns the precalculated potential complements based on the pathway and the seed complementarity approaches. *microbetag *supports optional network clustering with manta. The annotated network can then be parsed into Cytoscape using the MGG app.

As shown in Fig. 8, the *microbetag* workflow expects an abundance table representing either amplicon or shotgun data. If a microbial network is already available, the user may provide it too as input. The *microbetag* workflow will first map the taxa present in the abundance table to their corresponding GTDB representative genomes if that is possible, i.e., in case the taxonomy provided does reach the species or the strain level (see “Taxonomy schemes and genome assignment”). If a network is not provided, *microbetag* will build one using FlashWeave. Then, the abundance table will be used for a literature-based annotation using FAPROTAX. This is the only annotation step that is *microbetagDB*-independent within the web-service workflow. The nodes of the network will be further annotated with phenotypic traits based on *phenotrex* predictions [57]. Edges linking taxa assigned to the species or strain level will be annotated with pathway and seed complementarities and seed scores. Last, network clustering will be performed with manta, assigning each node to a cluster. The annotated network is then returned in a .cx2 format. The user may skip any of these annotation steps if not needed for their analysis.

### Taxonomy schemes and genome assignment

*Microbetag*’s on-the-fly version needs to map the taxonomy of an entry in the abundance table to its corresponding NCBI Taxonomy Id and, if available, its closest GTDB representative genome(s). Several GTDB representative genomes may map to the same NCBI Taxonomy Id. Two well established taxonomy schemes are supported: the GTDB [58] that is being broadly used for bins and/or MAGs (Metagenome Assembled Genomes) taxonomic classification and the Silva database [59] that is widely used in amplicon studies. Both taxonomy schemes link their taxonomies to NCBI Taxonomy IDs [60]. In case Silva, GTDB, or the GTDB-specific 16S rRNA database applied in the *microbetag* preprocessing tool (see “microbetag preprocessing” section) is used, *microbetag* requires an exact taxonomy match to assign a genome to the sequence/taxonomy under study. In case neither of those taxonomies is used, and the abundance table contains less than 1000 taxa, *microbetag* maps the user-provided taxonomies to the NCBI Taxonomy. To this end, *microbetag* makes use of the *fuzzywuzzy* library (v. 0.18.0) that implements the Levenshtein distance metric to get the closest NCBI taxon name and thus its corresponding NCBI Taxonomy Id; a high similarity score is used (90) to avoid false positives. Also, using the nodes dump file of NCBI Taxonomy, *microbetag* may retrieve the child taxa of a taxon in user data, along with their corresponding NCBI Taxonomy Ids, if requested by the user. If the user provides their abundance table with taxonomies already mapped to the GTDB taxonomy, *microbetag* will efficiently match them to corresponding entries in *microbetagDB* and return their associated annotations.

### Network inference

When a microbial network is not provided by the user, *microbetag* relies on FlashWeave (v. 0.19.2) [8] to build one on the fly. *microbetag* supports the annotation of networks built from any algorithm/software, in any format Cytoscape can load.

### *microbetag* preprocessing

To aid the user to map their sequences to the GTDB taxonomy, DADA2-formatted 16S rRNA gene sequences for both bacteria and archaea [61] were used to train the IDTAXA classifier of the DECIPHER package (v. 2.14.0) [62] and are available through the *microbetag* preprocessing Docker image. Likewise, when the abundance table consists of more than 1000 taxa and/or the taxonomy scheme is not among those that are automatically mapped, providing a network as an input is mandatory. The *microbetag* preprocessing Docker image also supports the inference of a network using FlashWeave.

### Literature-based node annotation

Using a set of Tara Ocean samples [63], FAPROTAX [64] estimates the functional potential of the bacterial and archaeal communities, by classifying each taxonomic unit into functional group(s) based on the current literature, descriptions of cultured representatives, and/or manuals of systematic microbiology. In this manually curated approach, a taxon is associated with a function only if all the cultured species within the taxon have been shown to exhibit that function. In the version *microbetag* makes use of, FAPROTAX (v. 1.2.4) includes more than 80 functions based on 7600 functional annotations and covers more than 4600 taxa. Contrary to gene-content-based approaches, e.g., PICRUSt2 [65], FAPROTAX estimates metabolic phenotypes based on experimental evidence. *microbetag* invokes the accompanying script of FAPROTAX and converts the taxonomic microbial community profile of the samples included in the user’s abundance table or of the taxa present in the provided network, into putative functional profiles. Then, it parses FAPROTAX’s sub-tables to annotate each taxonomic unit present in the user’s data with all the functions for which they had a hit. FAPROTAX annotations are not part of the *microbetagDB* but are computed on the fly.

### Genome-based node annotation

phenDB [57] is a publicly available resource that supports the analysis of bacterial (meta)genomes to identify distinct functional traits, e.g., whether a species is producing butanol or has a halophilic lifestyle. It relies on support vector machines (SVM) trained with manually curated datasets based on gene presence/absence patterns for trait prediction. More specifically, the model for a particular trait is trained using a collection of EggNOG annotated genomes [66] where the knowledge of whether that trait is present or absent among its members is available. These models (classifiers) are used to predict presence/absence of their corresponding traits in non-studied species.

**Fig. 9:**
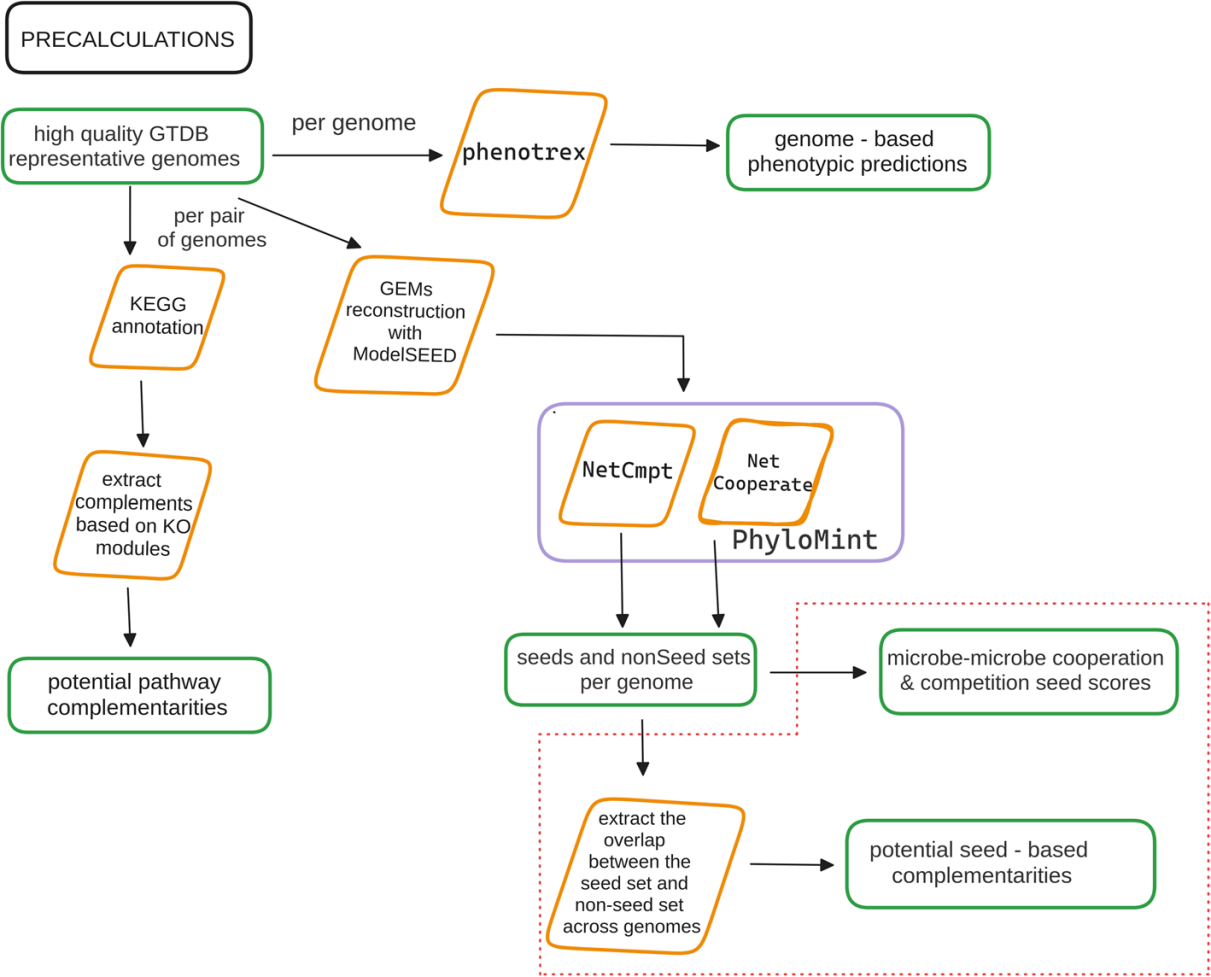
Diagram of *microbetag* precalculations. Current on-the-fly version GTDB v207 representative genomes were filtered and for those of high quality, 33 phenotypic traits were predicted using phenotrex. To this end, models were re-trained to sync with version 5.0 of EggNOG. To implement the pathway complementarity feature of *microbetag*, genomes are annotated with KEGG ORTHOLOGY terms and the KO terms related to KEGG modules were linked to the *alternatives* (see “Pathway complementarity”). For each genome, incomplete alternatives were complemented by identifying potential donor genomes carrying the missing term(s). Similarly, for the seed complementarity case, a metabolic network for each genome was reconstructed using ModelSEEDpy, and seed and non-seed sets of each genome were extracted and the MI_*Complementarity*_ and *MI*_*Competition*_ metabolic indices were calculated using an in-house, modified version of PhyloMInt. Based on the seed set of each genome and the non-seed sets of all the others, potential seed complementarities and scores can be extracted for all pairwise combinations (dotted box)

For *microbetagDB*, classifiers were re-trained using the genomes provided by phenDB for each trait to sync with the latest version of EggNOG (v. 5.0.0) [66] and the *phenotrex* (v. 0.6.0) [57] software tool. Genomes were downloaded from NCBI using the Batch Entrez program. Then, *genotype* files were produced for all the high-quality GTDB representative genomes. Each model was then used against all the GTDB *genotype* files to annotate each with the presence or the absence of the trait. A list of all the phenotypic traits tested for the genomes present in *microbetagDB* is available on *microbetag*’s documentation site. The updated models are also available. Figure 9 summarizes these precalculation steps. An exhaustive list of the traits whose presence or absence is predicted for each genome is available in Additional file 2: Table S1.

### Pathway complementarity

**Fig. 10:**
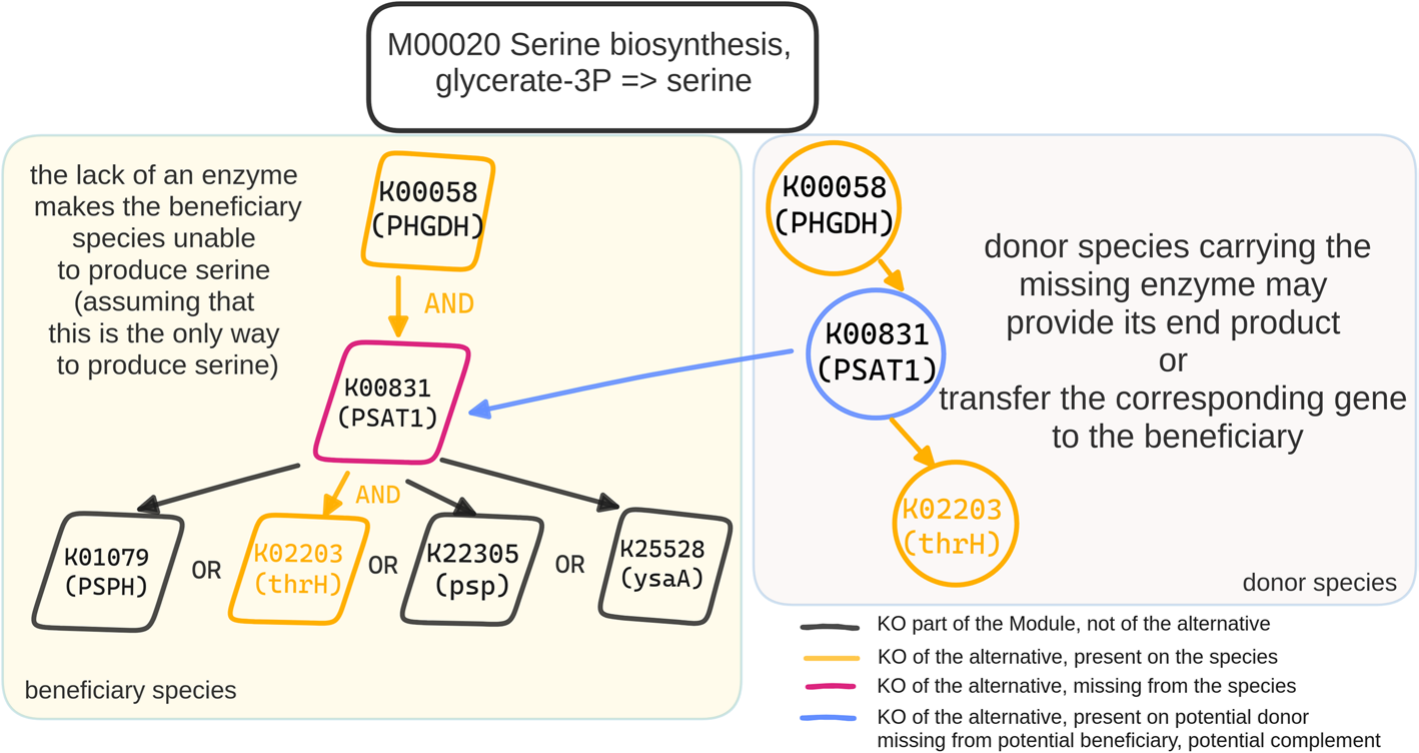
Pathway complementarity approach. The high-quality GTDB genomes were annotated with KEGG ORTHOLOGY (KO) terms. All the possible ways a donor species could “fill” a beneficiary’s incomplete KEGG module were calculated. In this case, there are four unique ways to complete the serine biosynthesis module; in all of them K00831 is required, which is missing from the beneficiary species that supports two out of the three steps of the module’s definition. The enzyme linked to KOO831 in the donor species completes the glycerate-3P to serine biosynthesis pathway in the beneficiary

To infer potential pathway complementarities, we rely on the modules in the KEGG MODULES database [28]. A KEGG module is defined as a functional unit within the KEGG framework that represents a set of enzymes and reactions involved in a specific biological process or pathway [28]. Such a unit consists of several *steps*, each of which may have more than one molecular way to occur (Fig. 10). A module’s definition is a logical expression and consists of KEGG ORTHOLOGY terms (KOs) that may be coupled with one another as (a) connected steps of the pathway, (b) parts of a molecular complex, (c) alternatives of the same step, and (d) optional entities of a complex. Both (a) and (b) cases should be considered as the AND logical operator, while (c) can be represented by the OR operator (Fig. 8). Given a module’s definition, we will consider as an *alternative* any subset of the KO terms mentioned in the definition that has exactly one way to perform each step, provided that all the steps of the module are covered. We define a genome as having a *complete* module only if all the KOs of at least one alternative are present in it. In Additional file 1: Supplementary information, we show an example of a module along with its alternatives. Within this framework, kofamscan (v. 1.3.0) [67] was used to annotate the 16,900 high-quality GTDB representative genomes for which a.faa file was available with KOs [68]. The KOs of each genome were then mapped to their corresponding KEGG modules; a KO term may map to more than one module (1:*n*).

All module definitions were retrieved using the KEGG API and parsed to enumerate their alternatives. Each pair of the KEGG annotated genomes was then investigated for potential pathway complementarities, i.e., whether a genome lacking a number of KOs (*genome*_*A*_) could benefit from another species’ genome(s) (*genome*_*B*_) to complete its module (*module*_*x*_). In that case, *genome*_*B*_ does not necessarily have a complete alternative of *module*_*x*_; as long as it has the missing KOs that *genome*_*A*_ needs to complete an alternative of it, *genome*_*B*_ potentially complements *genome*_*A*_ with respect to *module*_*x*_.

Thanks to the graphical user interface (GUI) of the KEGG pathway map viewer [69, 70], each complementarity can be visualized as part of a KEGG metabolic map. In this case, the KOs contributed by the donor are shown in blue-green whereas those coming from the beneficiary genome are colored in red.

*microbetag* annotates the edges of a microbial network by identifying pairs where both taxa map to an annotated genome present in *microbetagDB*. Since microbial networks are usually undirected, both nodes of an association are considered as potential donors and beneficiary species. When more than one GTDB representative genome maps to the same NCBI Taxonomy Id, all the possible genome combinations are considered. Finally, two directed edges are added for such taxon pairs in the annotated network: one considering *species*_*A*_ as the potential beneficiary and *species*_*B*_ as the potential donor species, and one vice versa.

### Seed scores and complementarity

Based on the seed set of a species, several indices estimating the metabolic interplay between two or more species have been suggested. The Metabolic Complementarity Index (*MI*_*Complementarity*_) measures the degree to which two microbial species can mutually assist each other by complementing each other’s biosynthetic capabilities. As described in [27], it is defined as the proportion of seed compounds of a species A that can be synthesized by the metabolic network of another species B, i.e., they are not seeds for species *B*. We will call this set the *non-seed set*, which represents the compounds the metabolic network can produce on its own. *MI*_*Complementarity*_ offers an upper bound assessment of the potential for cross-feeding interactions between two species.

Seeds might be independent, i.e., they cannot be produced by any other biochemical reaction in the metabolic network, or they can be interdependent, forming groups of seed compounds. Therefore, the size of each of the strongly connected components of the network can be used as the confidence score (*C*) for a compound being a seed. The Metabolic Competition Index (*MI*_*Competition*_) is defined as the intersection of the seed set of species A with the one of species *B*, normalized by the weighted sum of the confidence score (*C*). *MI*_*Competition*_ represents the similarity in two species’ nutritional profiles. This index establishes an upper limit on the level of competition that one species may face from another.

Those indices have been described and implemented in the NetCooperate [24] and NetCmpt [23] tools, respectively. We will be referring to those two indices as “seed scores”. Recently, the PhyloMint tool (v. 0.1.0) [27] was released, supporting the identification of seed and non-seed sets and enabling the subsequent calculation of the seed scores of metabolic networks in SBML format.

In the *microbetag* framework, seed scores were computed using metabolic networks derived from the high-quality GTDB representative genomes and the PhyloMint tool. Metabolic networks were reconstructed using the Model SEED pipeline [71] through its Python interface ModelSEEDpy (v. 0.4.2). The latter requires RAST annotated genomes [72]. If available through the PATRIC database [56], annotations were retrieved from PATRIC, otherwise, RAST annotation was performed through RASTtk (v. 1.3.0) [73]. Since PhyloMInt did not consider reaction reversibility, a modified version of the tool was adapted for use in *microbetag*.

**Fig. 11:**
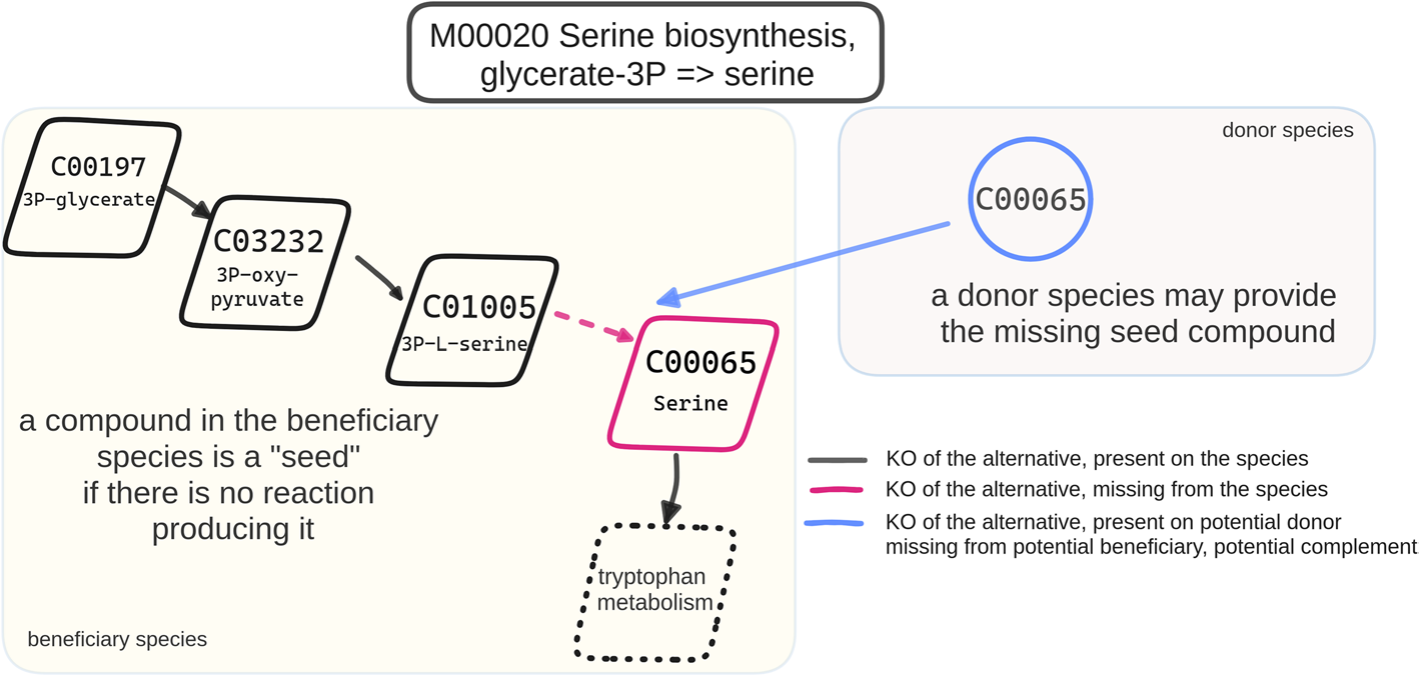
Seed complementarity. Seeds are compounds that, if provided exogenously, enable the beneficiary to produce every metabolite in its metabolic network. Such seed compounds can be obtained from the environment or through cross-feeding. In the case shown here, species A (beneficiary species) requires serine but cannot produce it on its own. However, a potential donor species B may provide it.

Seed and non-seed sets for the PATRIC annotated genomes were identified and stored so the on-the-fly version of *microbetag* can compute the overlap among all the pairwise combinations of the genomes mapped to a user’s dataset. Seed and non-seed compounds of each metabolic network are mapped to their corresponding KO terms (as substrates or products of their reactions) and those related to a KEGG MODULE are considered further. Focusing on the KEGG MODULE-related KO terms as terms of interest, the overlap of *seed set*_*speciesA*_ with the *non seed set*_*speciesB*_ is retrieved (Fig. 11). Edges of microbial networks where both taxa have been mapped to at least one GTDB genome are further annotated by listing all the KEGG maps for which there is at least one seed compound of use to the beneficiary species.

### Network clustering

*Manta* [15] is a heuristic network clustering algorithm that clusters nodes within weighted networks effectively, leveraging the presence of negative edges and discerning between weak and strong cluster assignments. *microbetag* invokes *manta* (edited version of v. 1.1.1) to cluster the microbial network. In case *manta* was executed, the annotated network inherits the layout that *manta* returns. However, as mentioned below, the microbetag GUI (MGG) can work with clusters resulting from any network clustering algorithm if it meets the expected output format.

### The MGG Cytoscape app

We developed a Cytoscape app to enable a straightforward, user-friendly way to perform the *microbetag* workflow and visualize *microbetag*-annotated networks. The *microbetag* GUI (*MGG*) app was built based on the source code of scNetViz [74] (forked from commit 336ac63). Under the Apps tab in Cytoscape, the app adds two menu entries: *MGG* and *MGG enrichment*. The first supports data import and on-the-fly execution, while the latter enables enrichment analysis once a *microbetag*-annotated network is loaded. Through the *MGG* entry, users can also apply the *MGG* visual style to a network. When applied, nodes are colored according to the level of their *microbetag* taxonomic assignment, with annotated nodes highlighted in green. Edges are colored blue for positive weights and dark pink for negative ones. All colors are designed to be color-blind friendly. Black directed edges represent pathway and/or seed complementarities. Through the *MGG* entry, users can also launch the *MGG result panel*—a two-tab panel that appears on the right side of the Cytoscape interface, with one tab focused on nodes and the other on edges. Network nodes and edges can be filtered based on their *microbetag* annotations, which can be inspected individually or in bulk. For more information about *MGG*’s features and usage, we refer readers to the relevant tabs on *microbetag*'s documentation page.

### Groups of phenotypic traits

Phenotypic traits returned from FAPROTAX and phenDB-like annotation steps are organized into biologically meaningful groups. The main groups supported are related to (a) the lifestyle of a species, for example, being halophilic or thermophilic, (b) the biogeochemical processes that are linked to the metabolic potential of a species, for example, nitrite-oxidizing bacteria (NOB) bacteria, and (c) important metabolites a species is predicted to produce, e.g., butanol. The aim of these groups is to facilitate filtering of the taxa present. Enrichment analysis for traits in such groups (e.g., based on the clusters identified by an algorithm like manta) can be performed through the *MGG* Cytoscape app.

### Software architecture

*Microbetag*’s pipeline is available as a Python package. To deploy the on-the-fly version, a Docker-based application was built. We deployed the *microbetag* application using Docker containers [75] (v24.0.2) managed by Docker Compose (see Additional file 1: Fig. S7). Docker Compose is a tool for defining and running multi-container Docker applications using a YAML file to configure the services required for the application. Containers of three Docker images are being used simultaneously: (a) a MySQL database including the *microbetagDB*, (b) a nginx [76] web server, and (c) the application itself, including the API and the *microbetag* workflow. Gunicorn (20.1.0) was used to build an application server which communicates with the web server using the Web Server Gateway Interface (WSGI) protocol and handles incoming HTTP requests. *microbetag* is implemented as a Flask application (v2.3.2); Flask is a micro web framework for developing Python web applications and RESTful APIs. The API has a route for performing the *microbetag* workflow, either through any Python console or the Cytoscape MGG app, but also several other routes that enable quick and easy access to the *microbetagDB* content, i.e., the genomes present, their phenotypic traits predicted based on genome annotations, pathway and seed complementarities among specific genomes or NCBI Taxonomy Ids, and their corresponding seed scores if available. A thorough description of the microbetag API is available at the documentation web site. The source code of the *microbetag* web service is available on GitHub (see Code availability).

## Supplementary Information

Additional file 1: Fig. S1 *Variovorax *and its closest neighbors. *Variovorax *annotations are shown in the node CyPanel. Fig. S2. Thiamine metabolism–related seed complementarities observed between*Variovorax*and its first neighboringstrains. Fig. S3. Biotin metabolism–related seed complements between *Variovorax*and its neighboring strains. Fig. S4. *microbetag*-annotated network of *Variovorax*and its first neighboring taxa from the Hessler et al.study. Fig. S5. Edge panel on *MGG* Cytoscape app from the *microbetag*-annotated network of the Cabrera et al.study. Fig. S6. Colored pantothenate and CoA biosynthesis KEGG map based on the pathway complementarity between *F. plautii *and *E. lenta*. Fig. S7. *microbetag* software ecosystem architecture.

Additional file 2: Table S1. Genome-based phenotypic traits predicted in the framework of *microbetag*. Table S2. Seed complementarity predictions from *microbetag*for *Variovorax *and its neighboring taxa, as described in Hessler et al.based on the on-the-fly *microbetag*version. Table S3. Seed complementarity predictions from *microbetag *for*Variovorax *and its neighboring taxa, as described in Hessler et al.based on the local *microbetag* version. Table S4. Thiamine-related potential seed complementarities among Variovorax neighboring taxa. Table S5. *Variovorax *genomes present on *microbetagDB* and their corresponding complete/incomplete presence of the pantothenate-related KEGG modules. Table S6. Enrichment/depletion findings on the subgingival plaque data.

## Data Availability

- *microbetag* pipeline (Python library), database, preprocessing modules and the documentation web site ([https://microbetag.readthedocs.io](https://microbetag.readthedocs.io)) are available on GitHub ([https://github.com/msysbio/microbetag](https://github.com/msysbio/microbetag)) [77] under GNU General Public License 3 and on Zenodo ([10.5281/zenodo.15619540](10.5281/zenodo.15619540)) [78]. - *microbetag* web application source code is available on GitHub ([https://github.com/msysbio/microbetagApp-public](https://github.com/msysbio/microbetagApp-public)) [79] under GNU General Public License 3, and on Zenodo ([10.5281/zenodo.16905743](10.5281/zenodo.16905743)) [80]. - *MGG* CytoscapeApp source code is available on GitHub ([https://github.com/msysbio/mgg](https://github.com/msysbio/mgg)) under Apache License 2.0 [81] and on Zenodo ([10.5281/zenodo.16905768](10.5281/zenodo.16905768)) [82]. - Raw sequences for the use case: The sequencing datasets generated by Cabrera et al. are available in the European Nucleotide Archive (ENA) repositories: - PRJEB29350 ([https://www.ncbi.nlm.nih.gov/bioproject/513540](https://www.ncbi.nlm.nih.gov/bioproject/513540)) [83] - PRJNA279279 ([https://www.ncbi.nlm.nih.gov/bioproject/PRJNA279279](https://www.ncbi.nlm.nih.gov/bioproject/PRJNA279279)) [84] - PRJNA629336 ([https://www.ncbi.nlm.nih.gov/bioproject/PRJNA629336](https://www.ncbi.nlm.nih.gov/bioproject/PRJNA629336)) [85] - Raw data for the test case: The sequencing datasets generated by Hessler et al. are available in the European Nucleotide Archive (ENA) repository: - PRJEB67393 ([https://www.ebi.ac.uk/ena/browser/view/PRJEB67393](https://www.ebi.ac.uk/ena/browser/view/PRJEB67393)) [86] - All files for the tutorials and the computing times mentioned can be found at microbetag’s GitHub repository [77]: [https://github.coma/msysbio/microbetag/tree/develop/docs/_static/download](https://github.com/msysbio/microbetag/tree/develop/docs/_static/download) Files with a.cys suffix correspond to Cytoscape sessions including *microbetag-* annotated networks.
