# Supplementary material for: microbetag: simplifying microbial network interpretation through annotation, enrichment tests, and metabolic complementarity analysis
Source: Genome Biol. 2025 Sep 22;26:292. doi: 10.1186/s13059-025-03769-2 (PMC12455805; doi:10.1186/s13059-025-03769-2)
Supplement: Supplementary file 1 — Additional file 1: Fig. S1 Variovorax and its closest neighbors. Variovorax annotations are shown in the node CyPanel. Fig. S2. Thiamine metabolism–related seed complementarities observed betweenVariovoraxand its first neighboringstrains. Fig. S3. Biotin metabolism–related seed complements between Variovoraxand its neighboring strains. Fig. S4. microbetag-annotated network of Variovoraxand its first neighboring taxa from the Hessler et al.study. Fig. S5. Edge panel on MGG Cytoscape app from the microbetag-annotated network of the Cabrera et al.study. Fig. S6. Colored pantothenate and CoA biosynthesis KEGG map based on the pathway complementarity between F. plautii and E. lenta. Fig. S7. microbetag software ecosystem architecture. [file 13059_2025_3769_MOESM1_ESM.docx]

# Supplementary Information

## Background on pathway and seed complementarity

For a genome to have a KEGG module *complete* means it provides at least one complete *alternative*. Alternatives are considered as the unique combinations of KOs that connect an input compound to an output compound of the module

For example, the definition of the D-Galacturonate degradation in Bacteria ([M00631)](https://www.genome.jp/dbget-bin/www_bget?M00631) is:

K01812 K00041 (K01685,K16849+K16850) K00874 (K01625,K17463)

Once breaking down, it leads to 4 alternative sets of KOs (pathways):

## 1. K01812 K00041 K01685 K00874 K01625 2. K01812 K00041 K16849+K16850 K00874 K01625 3. K01812 K00041 K01685 K00874 K17463 4. K01812 K00041 K16849+K16850 K00874 K17463

In alternatives two and four, the K16849+K16850 is a *complex*, meaning both KO terms are required for the step to be available.

In case of seed complementarity, in *microbetag* we focus on the effect that a metabolic exchange between two taxa might have if the seed of the beneficiary taxon is linked to a KEGG MODULE. Therefore, the KOs that were found linked to modules were mapped to ModelSEED ids. The initial seed and non-seed sets that were exported as sets of ModelSEED ids were then mapped to KOs too. When the non-seed set of a genome (donor) provides a seed related to a KEGG module to another genome (beneficiary), this is considered a potential metabolic interaction.

**
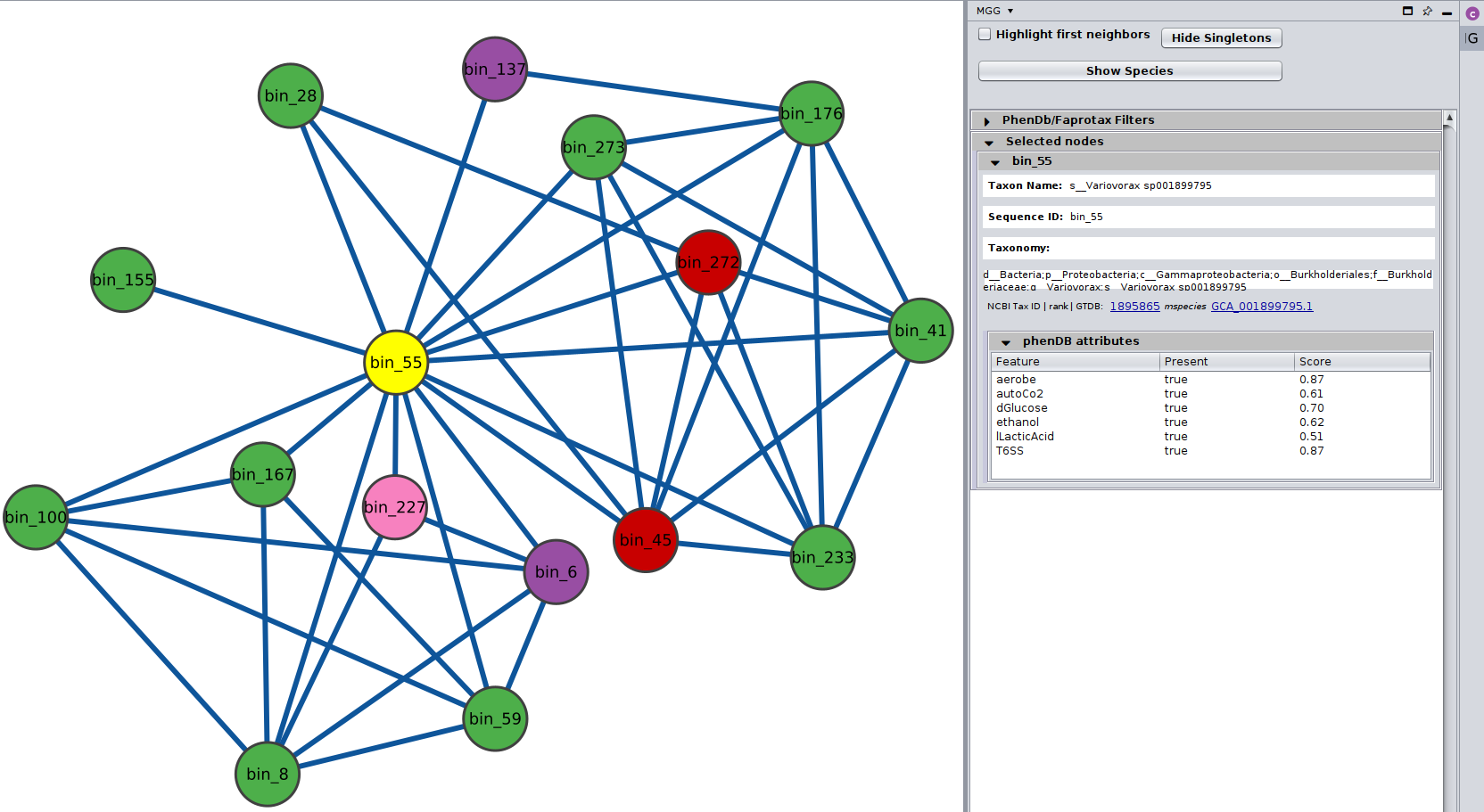
**

**Fig. S1**: *Variovorax* node (bin_55) and its neighbors annotated by microbetag. Only three of them were not mapped to a GTDB representative genome (pink and purple nodes denoting genus and family taxonomic levels accordingly). Green edges represent the positive association weights. The black edges represent pairwise seed complementarities and scores.


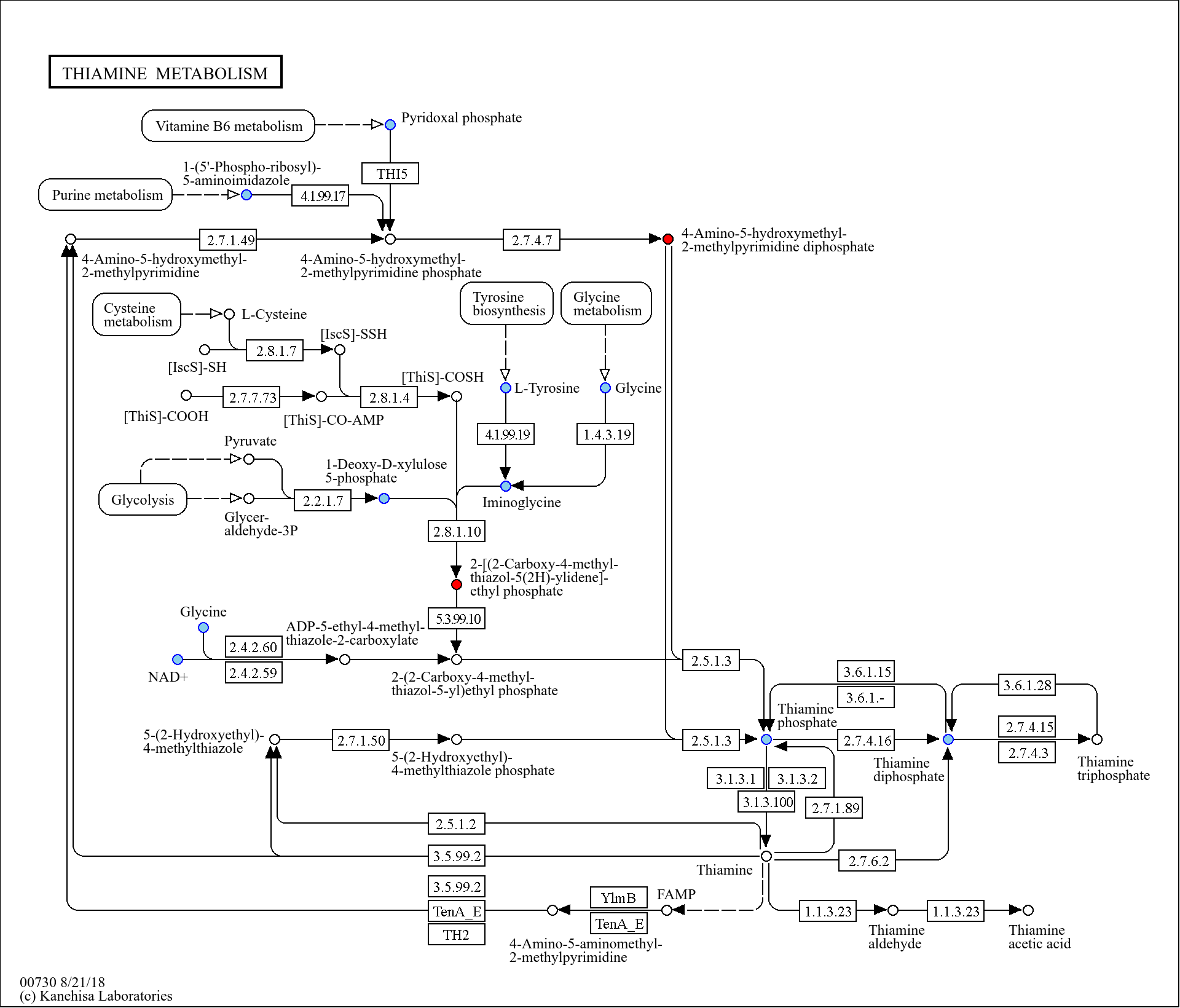


**Fig. S2**: Thiamine metabolism – related seed complementarities observed between *Variovorax* (donor) and its neighboring (beneficiary) strains. In 7 out of the 15 taxa found, *Variovorax* was found to be able to potentially provide at least one seed compound. In this case, seed complementarities for *Devosia*_A sp001899085 (bin_6) are shown.
C04752: 4-Amino-5-hydroxymethyl-2-methylpyrimidine diphosphate; C20246: cThz*-Pl.

**
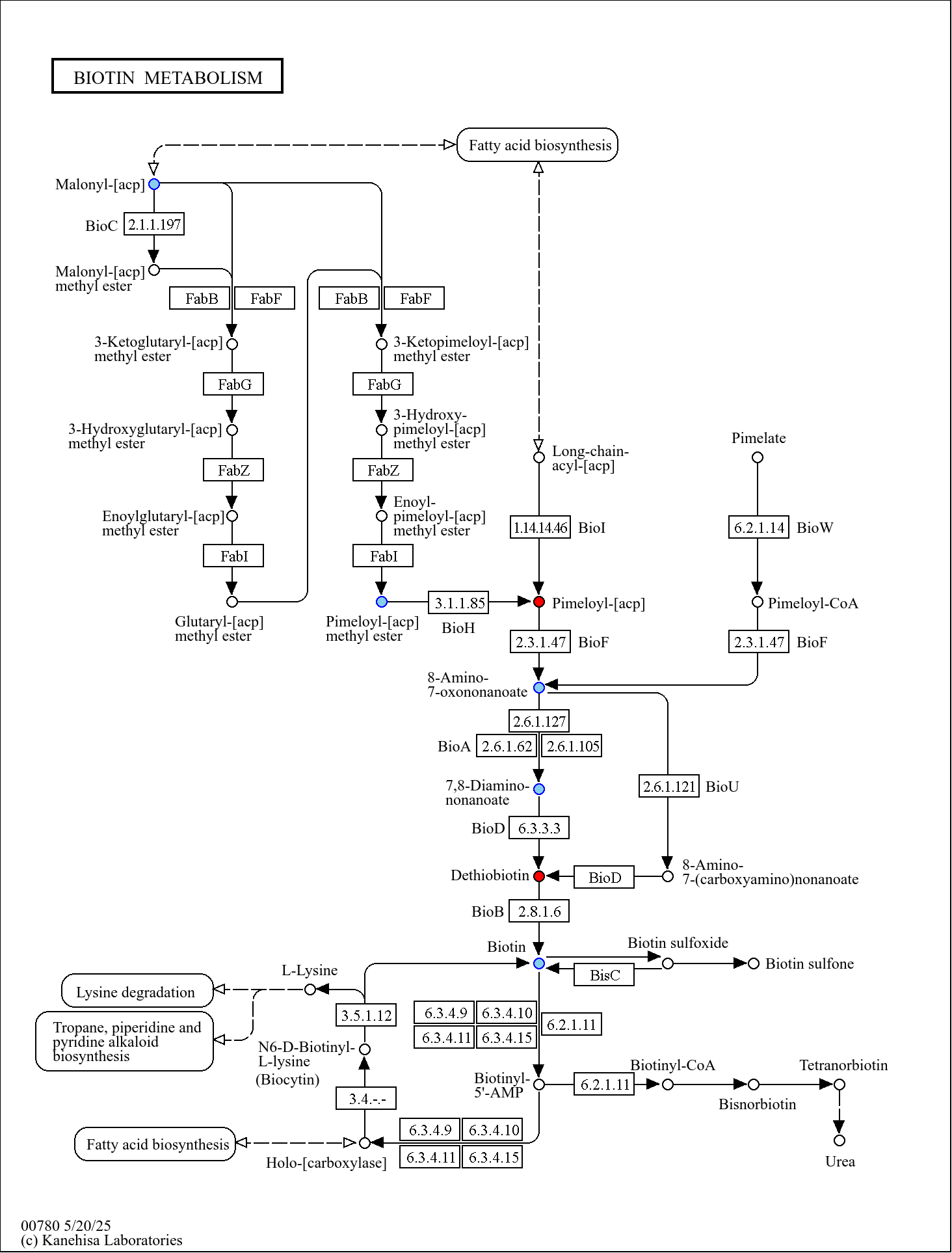
**

**Fig. S3:** Biotin metabolism – related seed complements between *Variovorax* (beneficiary) and its neighboring strains (donors). 11 out of the 15 neighboring taxa were found to be able to provide *Variovorax’*s missing compounds. In this case, seed complementarity findings from *Rhodoglobus* sp001725325 (bin_8) are shown: C19845: Pimeloyl-[acp]; C01909: Dethiobiotin.

**
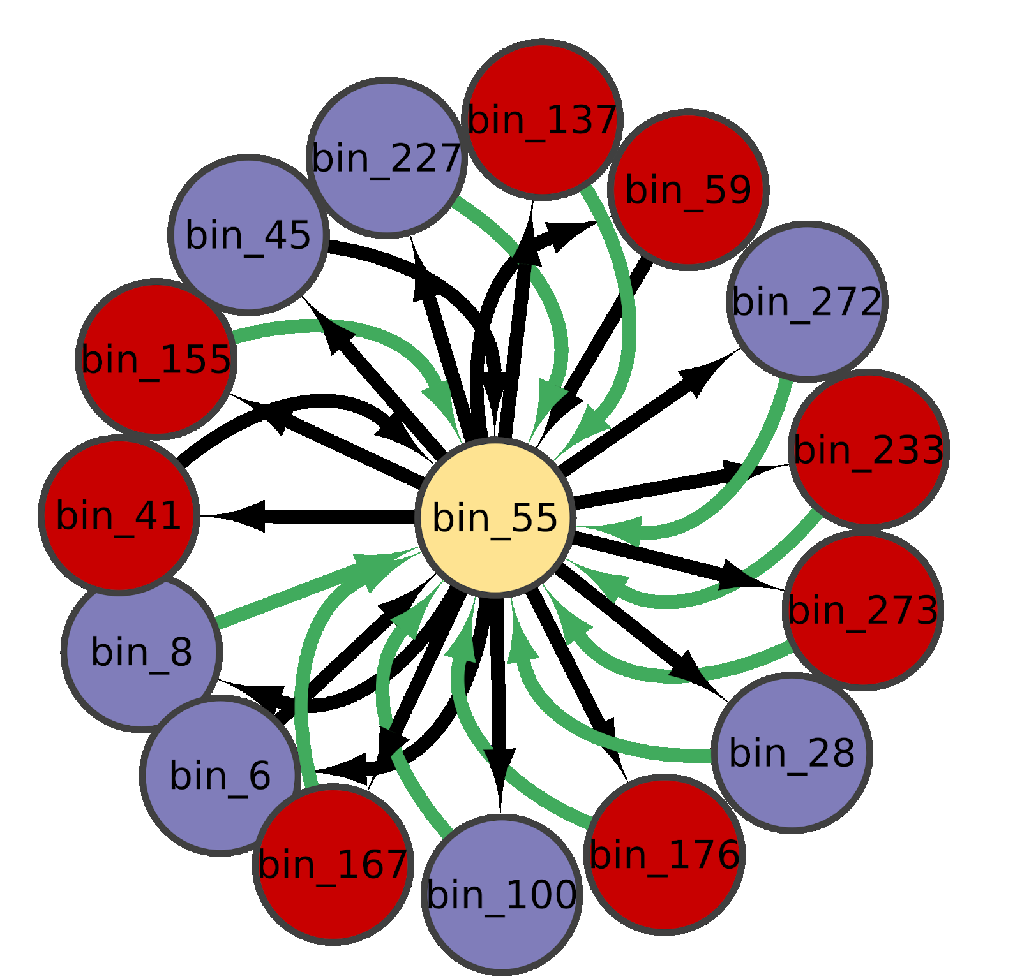
**

**Fig. S4:** *microbetag*-annotated network of *Variovorax* (bin_55) and its first neighboring taxa from the Hessler et al. (2023) study. Purple nodes represent taxa that were found to benefit from *Variovorax* with **thiamine**-related seeds, red nodes do not. Green edges were found to support **biotin** related seeds to *Variovorax.*

**
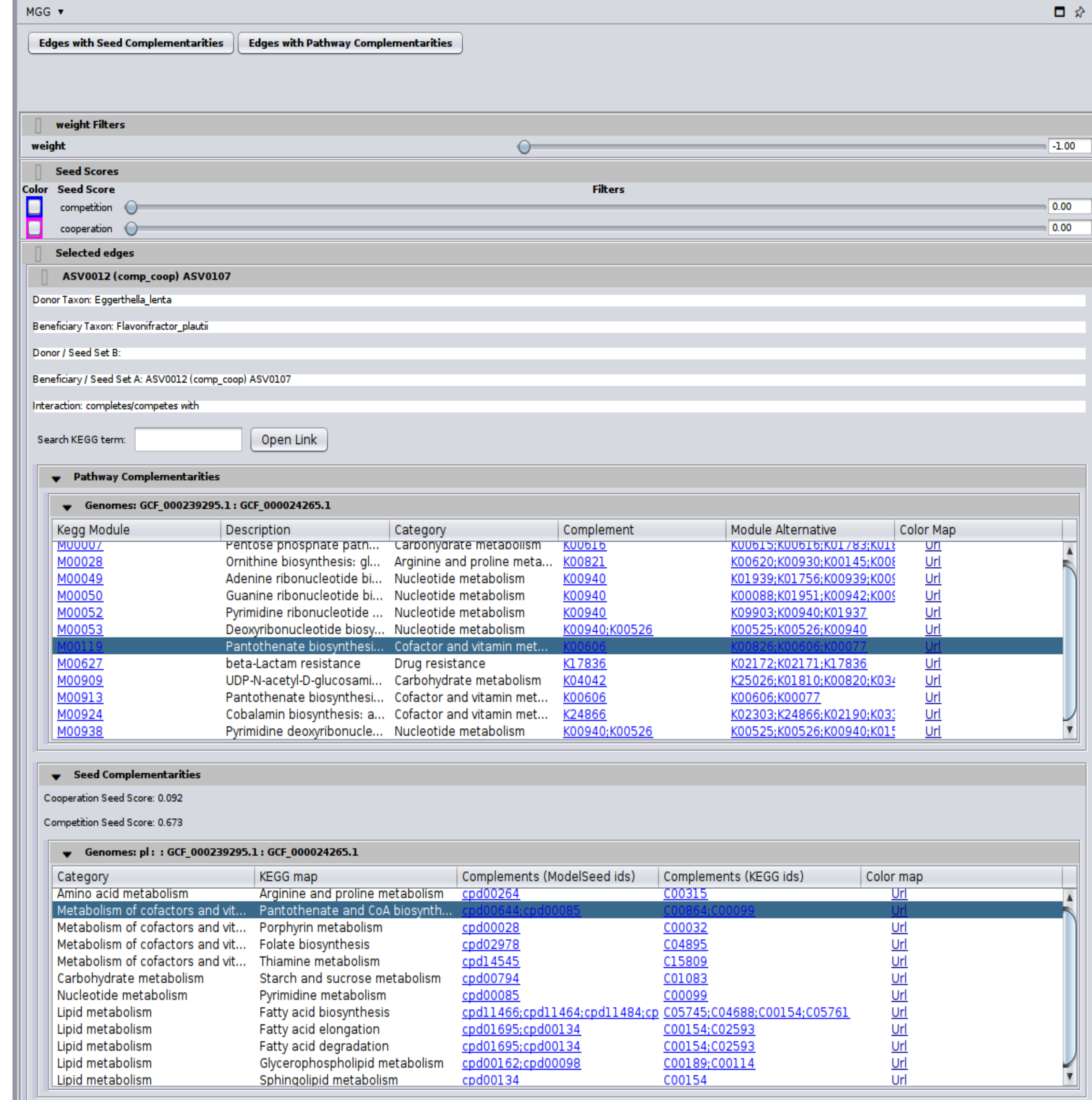
**

**Fig. S5**: Edge panel on *MGG* Cytoscape App from the *microbetag*-annotated network of the Cabrera et al. (2024) study (Use case 1), for the edge between ASV0012 (*Flavonifractor plautii*) and ASV0107 (*Eggerthella lenta*).

**
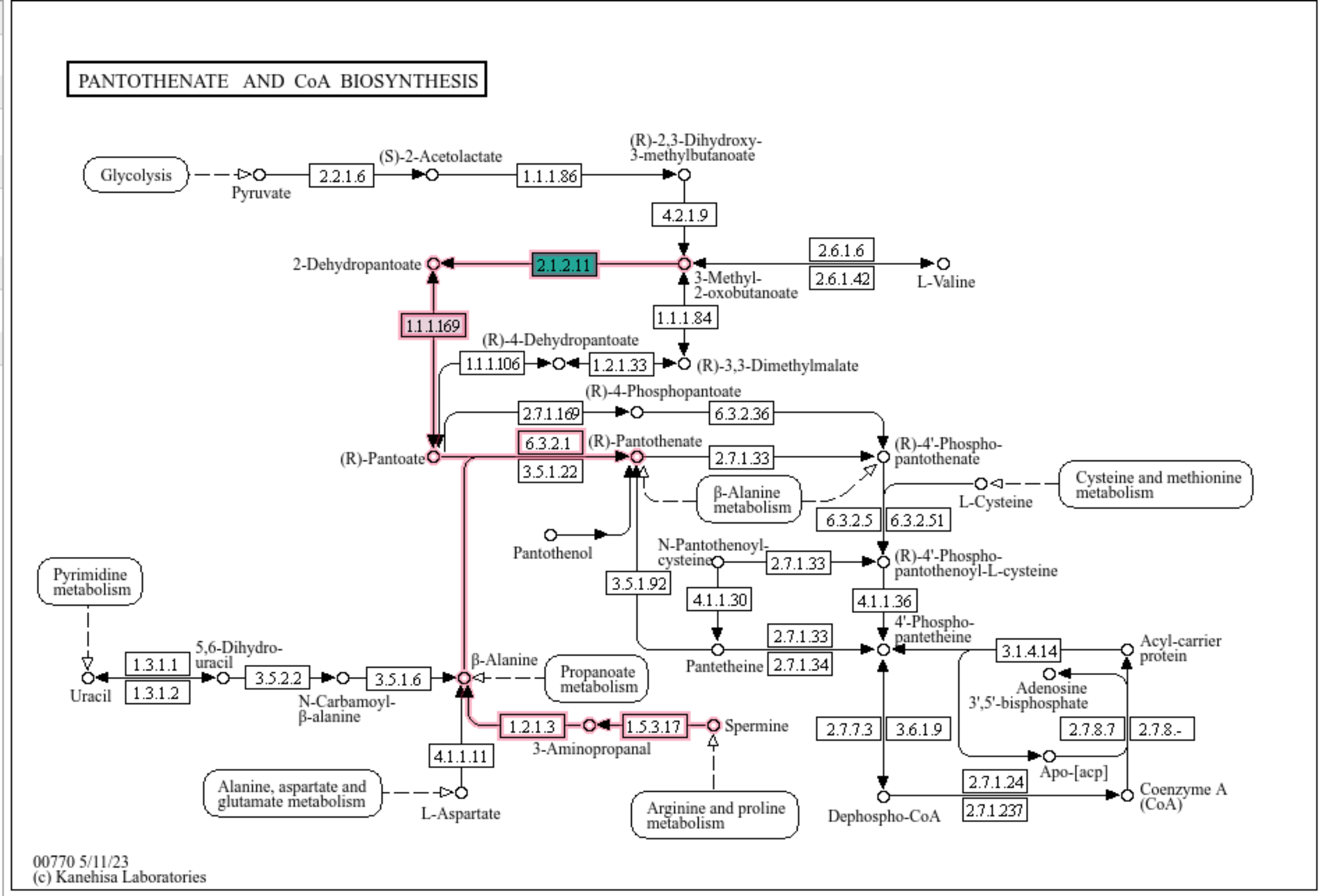
**

**Fig. S6**: Colored pantothenate and CoA biosynthesis KEGG map based on the pathway complementarity of microbetag suggesting that the missing enzyme (2.1.2.11) of the beneficiary species *F. plautii* could be acquired by the potential donor *Eggerthella lenta*.


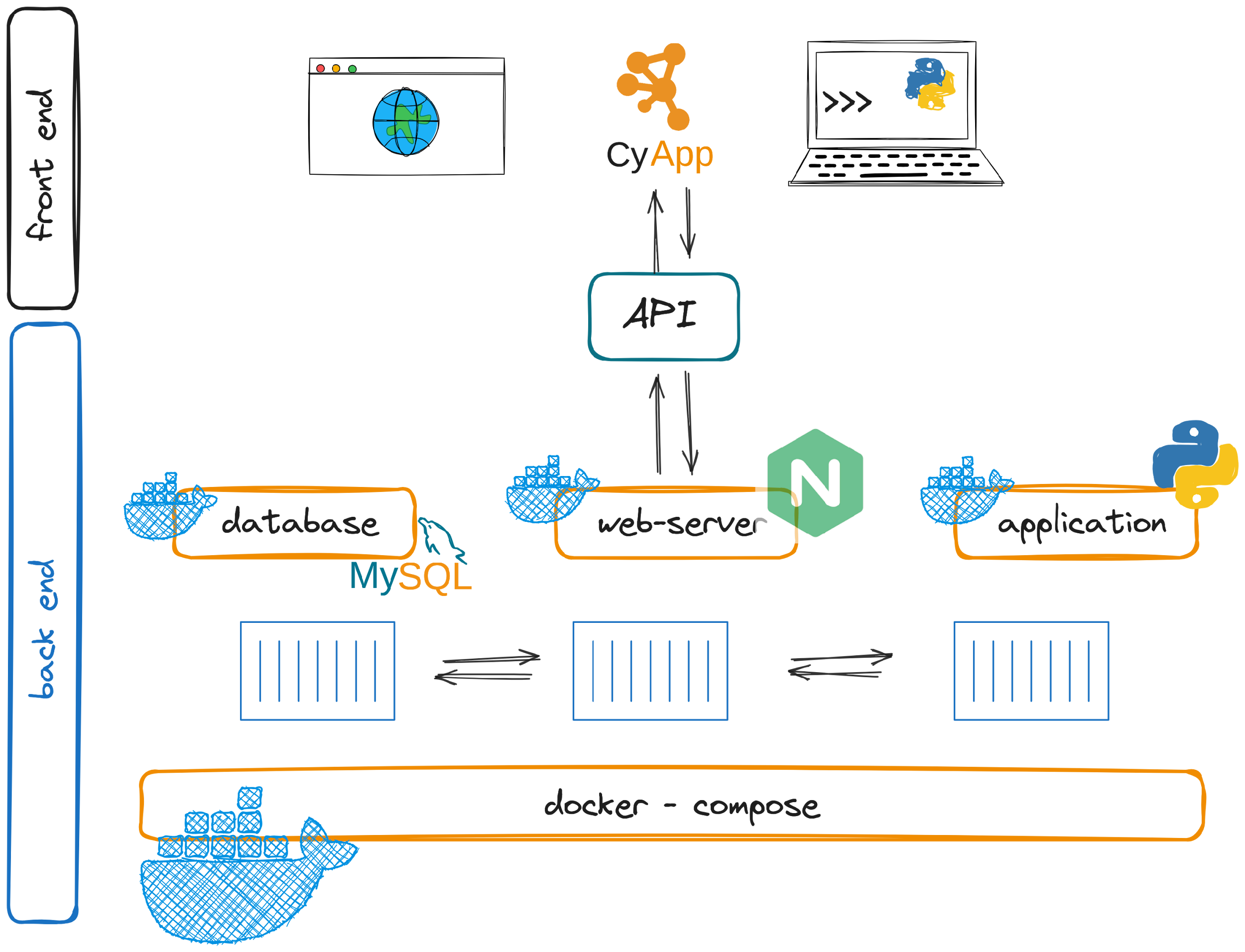


**Fig. S7**: *microbetag* software ecosystem. Three Docker containers are combined: a nginx web server connected to a MySQL database and the microbetag workflow. An API, as part of the last container, enables communication between the client (front) and the server side (back end). The content of the *microbetagDB* and the *microbetag* workflow are accessible through a web-browser, a terminal and the *MGG* CytoscapeApp.
